# Patterns in Benthic Microbial Community Structure Across Environmental Gradients in the Beaufort Sea Shelf and Slope

**DOI:** 10.3389/fmicb.2021.581124

**Published:** 2021-01-28

**Authors:** Alexis M. Walker, Mary Beth Leigh, Sarah L. Mincks

**Affiliations:** ^1^College of Fisheries and Ocean Sciences, University of Alaska Fairbanks, Fairbanks, AK, United States; ^2^Institute of Arctic Biology, University of Alaska Fairbanks, Fairbanks, AK, United States

**Keywords:** bacteria, archaea, marine sediment, arctic, microbial ecology, methane, anoxic

## Abstract

The paradigm of tight pelagic-benthic coupling in the Arctic suggests that current and future fluctuations in sea ice, primary production, and riverine input resulting from global climate change will have major impacts on benthic ecosystems. To understand how these changes will affect benthic ecosystem function, we must characterize diversity, spatial distribution, and community composition for all faunal components. Bacteria and archaea link the biotic and abiotic realms, playing important roles in organic matter (OM) decomposition, biogeochemical cycling, and contaminant degradation, yet sediment microbial communities have rarely been examined in the North American Arctic. Shifts in microbial community structure and composition occur with shifts in OM inputs and contaminant exposure, with implications for shifts in ecological function. Furthermore, the characterization of benthic microbial communities provides a foundation from which to build focused experimental research. We assessed diversity and community structure of benthic prokaryotes in the upper 1 cm of sediments in the southern Beaufort Sea (United States and Canada), and investigated environmental correlates of prokaryotic community structure over a broad spatial scale (spanning 1,229 km) at depths ranging from 17 to 1,200 m. Based on hierarchical clustering, we identified four prokaryotic assemblages from the 85 samples analyzed. Two were largely delineated by the markedly different environmental conditions in shallow shelf vs. upper continental slope sediments. A third assemblage was mainly comprised of operational taxonomic units (OTUs) shared between the shallow shelf and upper slope assemblages. The fourth assemblage corresponded to sediments receiving heavier OM loading, likely resulting in a shallower anoxic layer. These sites may also harbor microbial mats and/or methane seeps. Substructure within these assemblages generally reflected turnover along a longitudinal gradient, which may be related to the quantity and composition of OM deposited to the seafloor; bathymetry and the Mackenzie River were the two major factors influencing prokaryote distribution on this scale. In a broader geographical context, differences in prokaryotic community structure between the Beaufort Sea and Norwegian Arctic suggest that benthic microbes may reflect regional differences in the hydrography, biogeochemistry, and bathymetry of Arctic shelf systems.

## Introduction

The Arctic marine ecosystem is undergoing pronounced changes due to climbing atmospheric temperatures, occurring two to three times faster than the global average (ACIA, 2005; Walsh et al., 2011). Resulting shifts in sea-ice cover, primary production, and riverine input will likely affect the quality and quantity of organic material (OM) deposited to the seafloor and the tight pelagic-benthic coupling characteristic of Arctic ecosystems may emphasize the effects of these changes (Grebmeier, 2012; Kortsch et al., 2015). Impacts of climate change on Arctic benthic eukaryotes have varied among size classes, but benthic prokaryotic communities have not been described well enough to monitor change (Nelson et al., 2014; Kêdra et al., 2015; Renaud et al., 2019).

Benthic bacteria and archaea perform diverse metabolic functions that mediate biogeochemical processes, including degradation and early diagenesis of organic matter in marine surface sediments (Deming and Baross, 1993). Prokaryotes provide a link between the abiotic and biotic realms, such that prokaryotic community structure reflects environmental gradients in, for example, OM deposition. Studies conducted in the Norwegian Arctic suggest that prokaryotic community structure shifts with varying quality and quantity of OM inputs, with possible consequences for ecosystem function (Hoffmann et al., 2017; Braeckman et al., 2018). Certain taxonomic groups have been strongly correlated with environmental parameters, such as Chl-*a* and bathymetry, measured along natural environmental gradients (Bienhold et al., 2012; Jacob et al., 2013). Benthic prokaryotes are also key players in both the production and degradation of greenhouse gases such as methane and nitrous oxide and represent a first line of defense in remediating contamination by oil and other petroleum-based products (Boetius et al., 2000; Head et al., 2006; Casciotti and Buchwald, 2012). In some cases, these important functions are attributable to specific taxonomic groups (Kostka et al., 2011; Ruff et al., 2015; Otte et al., 2019). Thus, characterization of benthic prokaryotic community structure may yield valuable insights into ecosystem function in Arctic marine sediments, particularly those permeated with methane and subjected to active mineral resource exploration such as the Beaufort Sea sites examined here (Coffin et al., 2013; BOEM, 2019).

The Beaufort Sea is an Arctic marginal sea that crosses a border between the United States and Canada, stretching from Point Barrow (Alaska, United States) to Banks Island (Northwest Territories, Canada). Prior studies of benthic prokaryotes in the southern Beaufort Sea have focused on subsurface sediments (20–70 cm) to assess the influence of either methane or mud volcanoes on bacterial community structure (Hamdan et al., 2013; Kirchman et al., 2014; Treude et al., 2014; Lee et al., 2018). Communities in these deeper sediments typically differ substantially from the active surface layer (Inagaki et al., 2003; Fry et al., 2006; Roussel et al., 2009; Durbin and Teske, 2011; Jorgensen et al., 2012; Bienhold et al., 2016). Thus, there is no established baseline for benthic prokaryotes in southern Beaufort Sea surface sediments to date, and only limited data from deeper sediment horizons. Most information on Arctic benthic prokaryotes comes from the Norwegian Arctic, largely from deep-sea or fjord habitats, which differ from the Beaufort Sea in terms of primary production, terrestrial/riverine input, and bathymetry (Ravenschlag et al., 2001; Holmes et al., 2002; Carmack and Wassmann, 2006; Arnosti, 2008; Li et al., 2009; Jacob et al., 2013; Buttigieg and Ramette, 2015; Bienhold et al., 2016; Zeng et al., 2017).

**FIGURE 1:**
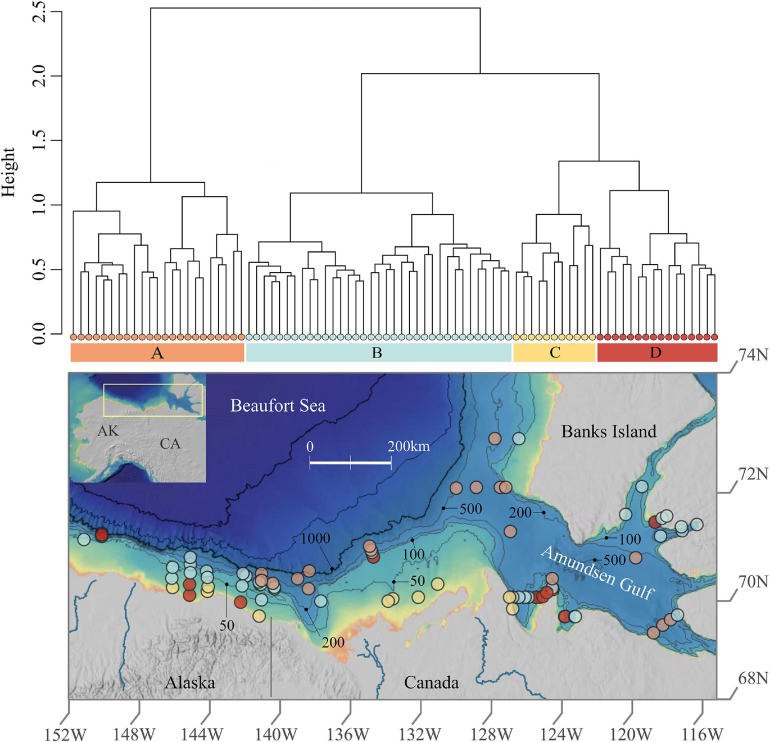
Prokaryotic cluster assemblages and spatial distribution in Beaufort Sea sediments. This figure shows the dendrogram of the four assemblages revealed via hierarchical clustering above the study area map which exhibits sample locations colored by assemblage. The map was created using ArcGIS online with the following layers: Northwest Territories, Esri, Garmin, FAO, NOAA, USGS, EPA, NRCan, and Parks Canada.

Across the southern Beaufort Sea, summer depth-stratified water-mass structure includes a fresher surface Polar Mixed Layer (PML; ∼0–50 m), the Pacific-influenced Arctic Halocline Layer (AHL; ∼50 – 200 m), and warmer, more saline Atlantic Water (AW) at depths greater than 200 m (Lansard et al., 2012; Miquel et al., 2015; Smoot and Hopcroft, 2017). Eastern Beaufort Sea shelf (< 100 m water depth) and slope habitats are particularly influenced by riverine input, including the vast Mackenzie River which is the fourth largest in the Arctic in terms of freshwater discharge (330 km^3^/year), and the largest in terms of sediment transport (124 × 10^6^ t/year) (Macdonald et al., 1998; Holmes et al., 2002; Rachold et al., 2004). The Mackenzie River primarily influences the water and sediment characteristics of the Canadian sector of the shelf and slope, and to a lesser extent the Amundsen Gulf and easternmost section of the Alaskan sector (Figure 1). The Mackenzie shelf functions as a vast estuary influenced by freshwater runoff, receiving inputs of both terrestrial and marine sources of organic matter (Carmack and Macdonald, 2002; Magen et al., 2010). The Amundsen Gulf, which connects the Canadian Arctic Archipelago with the southeast Beaufort Sea, is characterized by several peripheral bays, straights, and inlets and ringed by a narrow shelf surrounding a central basin (Stokes et al., 2006; Forest et al., 2010; Gamboa et al., 2017). Water mass properties in the Alaskan Beaufort Sea are influenced by warmer, more nutrient-rich waters entering from the Chukchi Sea and forming the Beaufort Shelf Jet, which flows due east from Barrow Canyon (Pickart, 2004; Weingartner et al., 2005; Dunton et al., 2006). These longitudinal environmental gradients from Point Barrow to Banks Island have been reflected in the benthic distributions of OM and certain epi-/infaunal groups (Naidu et al., 2000; Magen et al., 2010; Goñi et al., 2013; Bell et al., 2016). This benthic habitat may also be heavily influenced by methane derived from organic matter burial, mud volcanoes, subsurface permafrost, and/or gas hydrates (Paull et al., 2015; Brothers et al., 2016; Coffin et al., 2017; Lee et al., 2018; Retelletti Brogi et al., 2019).

We sought to describe prokaryote communities across this broad, heterogeneous benthic habitat, specifically by (1) assessing the diversity, structure, and composition of archaeal and bacterial communities in the upper 1 cm of sediments, and (2) investigating environmental correlates of prokaryote community structure over a broad area of the southern Beaufort Sea shelf and slope. Expanding knowledge of functional taxonomic groups of marine prokaryotes provides insight into this unstudied microbial habitat, and a framework for developing more targeted experimental studies in this dynamic polar environment (Fuhrman, 2009; Bier et al., 2015; Fuhrman et al., 2015).

## Materials and Methods

### Sample Collection

Our study area covers a broad section of the continental shelf and upper slope of the Alaskan and Canadian Beaufort Sea, from areas offshore of the Colville River in the west to Banks Island in the east, and into the Amundsen Gulf (Figure 1). A total of 85 sediment samples were collected opportunistically at water depths between 17 and 1,200 m from 2012 to 2014 as part of two international collaborative field programs: the US-Transboundary Fish and Lower Trophic Communities Project (USTB) and the Canadian Beaufort Regional Environmental Assessment Project (BREA). Fifteen sediment samples, 8 in 2012 and 7 in 2013, were collected using a 5 cm diameter sub-core from the upper 1 cm layer of a 0.25 m^2^ box core. In 2014, 70 sediment samples were collected using a 60-cc, 2.5 cm diameter, sterilized syringe from the upper 1 cm surface layer of a double van Veen grab (0.1 m^2^, USTB) or a 0.25 m^2^ box core (BREA). As preliminary analyses indicated that neither year or sampling gear were significant factors affecting prokaryotic community structure here, all samples were used in this study. The samples were immediately frozen at −20°C following collection aboard respective vessels, transported to the University of Alaska Fairbanks (UAF) Institute of Arctic Biology Genomics Core Laboratory, and then stored at −80°C. Environmental variables such as depth, bottom water temperature, and salinity were recorded from CTD profiles conducted at corresponding sampling locations (Eert et al., 2015; Niemi et al., 2015; Smoot and Hopcroft, 2017).

### Sediment Characteristics

We quantified several common indicators of quality, quantity, and origin of sediment OM including chlorophyll-*a* concentration (Chl-*a*), phaeopigment concentration (Phaeo), total organic carbon (TOC), total nitrogen (TN), ratio of carbon to nitrogen (C:N), and bulk sediment δ^13^C and δ^15^N. All parameters were measured using sediment samples taken from the upper 1 cm of the same box core or grab as the genomic samples from each sampling station. Chl-*a* and phaeopigment concentrations were measured fluorometrically (Arar and Collins, 1997). Briefly, samples were suspended in 5 ml 100% acetone, sonicated in an ice-water bath for 10 min, and allowed to extract overnight at −20°C. Samples were then centrifuged to remove sediment, and transferred to a clean test tube. Chl-*a* concentration was determined using a TD-700 fluorometer (Turner Designs). After recording fluorescence values, samples were acidified with HCl, and fluorescence readings were taken of the acidified samples to produce phaeopigment values. A standard curve produced using commercially available Chl-*a* standard was used to convert fluorescence readings into concentrations.

Stable isotope and elemental analysis were performed on freeze-dried sediment samples at the Alaska Stable Isotope Facility (ASIF). Data for USTB samples were generated and previously reported by Bell et al. (2016); additional samples were analyzed here using the same protocol. Stable isotope data, δ^13^C and δ^15^N, were generated using a ThermoFinnigan DeltaVPlus isotope ratio mass spectrometer with Pee Dee Belemite (PDB) and atmospheric nitrogen as standards for carbon and nitrogen, respectively. Percent carbon and nitrogen content was obtained using a Costech ESC 4010 elemental analyzer, and used to calculate TOC, TN, and C:N ratios.

Sediment porosity (Φ), i.e., volume of water within sediments (V_*w*_), was calculated as an indicator of sediment permeability/O_2_ penetration using Eq. 1 (Bennett and Lambert, 1971; Ullman and Aller, 1982). Sediment wet (W_*sw*_) and dry (W_*sd*_) weights were recorded before and after freeze-drying, and used to calculate the mass of water (Ww). Sediment density (ρ_*s*_) was held constant at 2.50 g/cm^3^, based on Coffin et al. (2017). Seawater density (ρ_*w*_) was calculated based on bottom-water temperatures and salinities recorded at sampled sites.

(1) $$\text{Porosity}\,\left(\phi \right)={\text{V}}_{\text{W}}=\frac{\frac{\left({\text{W}}_{\text{sw}}-{\text{W}}_{\text{sd}}\right)}{\rho_{\text{w}}}}{\left(\frac{{\text{W}}_{\text{sd}}}{\rho_{\text{s}}}\right)+\left(\frac{{\text{W}}_{\text{w}}}{\rho_{\text{w}}}\right)}$$

### Microbial Community Analyses

16S ribosomal (rRNA) gene amplicon sequencing was conducted on freeze-dried sediments in order to assess the diversity and taxonomic composition of prokaryotes. Total genomic DNA was extracted from sediment samples using the Qiagen PowerSoil kit, and revised forward (515FB) and reverse (806RB) primers from the Earth Microbiome Project (EMP) were used to amplify the V4 region of the 16S rRNA gene (Caporaso et al., 2011, 2012; Apprill et al., 2015; Parada et al., 2016). Library prep was conducted using iTru adapters for sequencing and a one-step PCR protocol with indexed primers, which is the current standard protocol used by the EMP (Caporaso et al., 2011, 2012; Apprill et al., 2015; Parada et al., 2016; Walters et al., 2016). Samples were sequenced on an Illumina MiSeq at the UAF Genomics Core Lab.

Raw sequences were de-multiplexed using the Mr. Demuxy package (Cock et al., 2009). Demultiplexed sequences were run with mothur v1.40.0 on a high performance-computing cluster through UAF Research Computing Systems using a modified MiSeq standard operating procedure (Schloss et al., 2009). Operational taxonomic units (OTUs) were clustered at 100% similarity using the OptiClust option in mothur, taxonomy was assigned to OTUs using the SILVA 132 mothur formatted reference database with a bootstrap cutoff of 100%, and the samples in the resulting OTU table were normalized to 30,000 sequences and converted to relative abundances (Wang et al., 2007; Glöckner et al., 2017; Westcott and Schloss, 2017; Edgar, 2018). This OTU table (relative abundance of sequence reads per OTU for each sample) was used to conduct analyses of diversity and community structure of prokaryotes and to assess correlations with environmental variables.

### Data Analyses

All statistical analyses were conducted using R, primarily using the vegan package (Oksanen, 2015; R Core Team, 2017). Diversity was quantified using the inverse Simpson index (1/λ) which reflects evenness and richness (Morris et al., 2014). Community structure was investigated via hierarchical clustering analysis with the Ward method (ward.D2) based on a Bray-Curtis dissimilarity derived from the OTU table. Cluster tests were used to assess the validity (heterogeneity, significance) and characteristics (silhouette, stability) of the hierarchical clusters. The heterogeneity of and significance between hierarchical clusters were investigated using Bray Curtis distances with the betadisper and adonis functions, respectively (Oksanen et al., 2017). OTUs that distinguished each cluster were identified via indicator taxa analysis using the indicspecies package (De Cáceres and Legendre, 2009; De Cáceres et al., 2010). The multipatt function was used to identify taxa specific to a single cluster, or indicative of combinations of clusters, with a significance of ≤0.001 and a strength of ≥0.900 (i.e., >90% probability of occurrence within a given cluster or combination of clusters (De Cáceres and Legendre, 2009). Given the complexity of the prokaryotic species concept and the lack of taxonomic resolution for many taxa, we used this indicator analysis to identify OTUs potentially indicative of certain habitat features and/or assemblages. We assessed the distribution of these indicator taxa at varying taxonomic levels, from phylum to genus, to characterize representative assemblages for each cluster.

Differences in the median values of environmental parameters among clusters were evaluated using Kruskal-Wallis tests followed by pairwise Wilcoxon Rank Sum tests with a Benjamini and Yekutieli (BY) correction (Yekutieli and Benjamini, 2002). Relationships between prokaryotic community structure and environmental variables were modeled using Constrained Analysis of Principle Coordinates, i.e., the capscale function in the R vegan package (Oksanen, 2015; R Core Team, 2017). The best model for capscale analyses was identified based on variance inflation factors for each environmental parameter and a forward and backward stepwise model selection via permutation tests on adjusted *R*^2^ and *P*-values using the ordistep function. The significance of the environmental correlates yielded via Constrained Analysis of Principle Coordinates was investigated in R using the adonis function; correlation strengths were calculated using the cor function (Oksanen et al., 2017). All bioinformatic and statistical scripts can be found here on GitHub^1^.

In order to put Beaufort Sea surface sediment microbes into a broader geographical context, we qualitatively compared the 10 most abundant taxa at class level with those reported for global benthic surface sediments, global deep-sea sediments, and Norwegian-Arctic sediments (Zinger et al., 2011; Bienhold et al., 2016). We refer to the Arctic microbiome published by Bienhold et al. (2016) as the Norwegian Arctic microbiome because all but one dataset included in that study was generated from the Norwegian Arctic (Bienhold et al., 2016).

## Results

### Prokaryotic Community Structure and Diversity

The total number of OTUs yielded was 295,115. No OTUs were removed (e.g., singletons or doubletons) because removals resulted in substantial changes in certain taxonomic groups (Supplementary Figure 1). Hierarchical clustering analysis identified four clusters, i.e., assemblages of prokaryotes which have significantly different (adonis: *F* = 12.6, *P* < 0.001) taxonomic composition (Figure 1). However, the silhouette test yielded an average cluster width of 0.11, indicating a high degree of similarity between clusters which suggests that the four assemblages may exist more so as a gradient rather than as discrete communities in particular locations. The number of samples within each cluster, herein referred to as assemblages A, B, C, and D, was as follows: A = 23, B = 35, C = 11, and D = 16. Assemblage A is most distinct, whereas C and D are most similar (Figure 1). The distribution of values for Inverse Simpson diversity differed for each assemblage, with the highest prokaryotic diversity exhibited in assemblage A, then B, D, and C.

**FIGURE 2:**
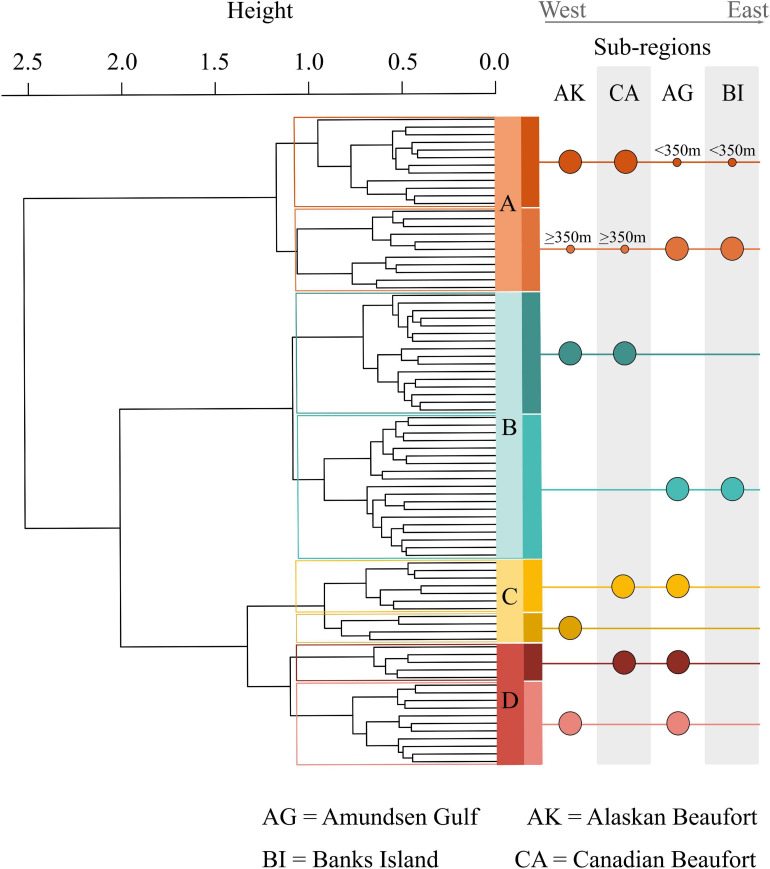
Substructure of prokaryotic assemblages. This dendrogram highlights the substructure within each assemblage and the differing east to west gradients reflected by the substructure. Assemblage A was divided by location and depth, with one sub-cluster dominated by Amundsen Gulf (AG) samples combined with broader Beaufort (AKCA) slope samples deeper than 350 m. The other assemblage A sub-cluster was dominated by broader Beaufort slope samples with AG samples shallower than 350 m. Assemblage B divided cleanly between broader Beaufort and Amundsen Gulf samples. Assemblage C divided between the west of the Mackenzie River, AK samples, and east of the Mackenzie River, CA Beaufort and AG samples. Assemblage D divided into combinations of AG samples with either AK or CA samples with no obvious demarcations.

Each assemblage is represented by sampling locations distributed across the study area (Figure 1), such that assemblages were not restricted to particular geographic areas that might reflect distinct environmental characteristics. However, when delving into the substructure of individual assemblages, patterns emerged at smaller spatial scales (Figure 2). Assemblage A contained two clusters, with one containing mostly Alaskan and Canadian Beaufort (AKCA) samples and some shallower (<350 m) Amundsen Gulf and Banks Island (AGBI) samples, and the other containing mostly AGBI samples with a few deeper (>350 m) AKCA samples. Assemblage B was clearly divided into AKCA and AGBI samples. Sub-structure for assemblage C was delineated by the Mackenzie River into the AK Beaufort shelf (west of the river) and the CA Beaufort with AG samples (east of the river). Assemblage D divides into two groups with no obvious geographical demarcations.

### Taxonomic Composition and Indicator Taxa

**FIGURE 3:**
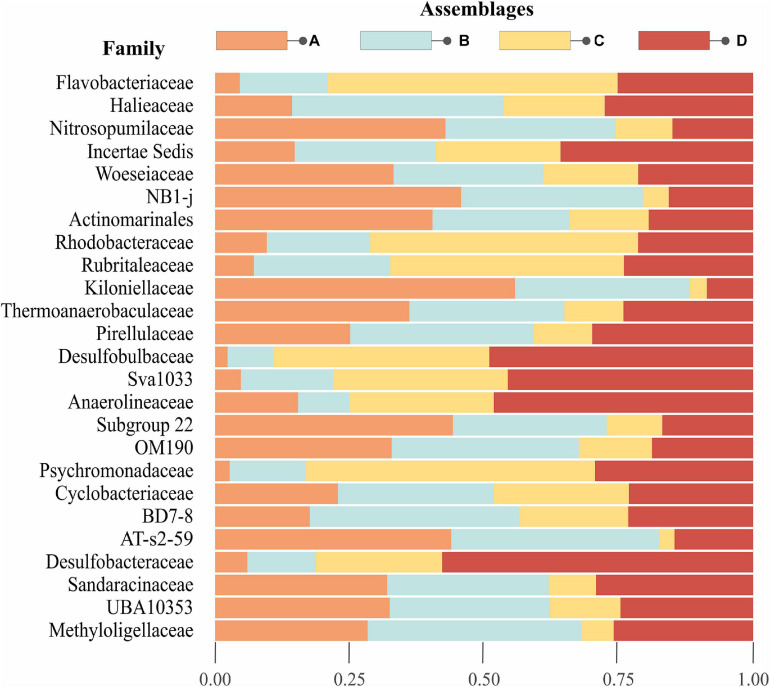
Top 25 abundant families in Beaufort Sea surface sediments. The stacked barplot illustrates the proportion of the top 25 families for each assemblage on the left-hand *y*-axis. The plot is arranged such that the most abundant taxa starts from the top and decreases toward the bottom. Note that Gammaproteobacteria Inc. sed., NB1-j, Actinomarinales, Sva1033, OM190, BD7-8, AT-s2-59, and UBA10353 are not classified to family, but order level.

There were 350 prokaryotic families represented in this dataset; we focused on the 25 most abundant families (or in some cases, orders) to assess broad-scale patterns in community structure and compare relative abundance of these dominant taxa among individual assemblages. Abundant taxa consisting of multiple unclassified OTUs within a class or phylum, specifically Alpha- and Gammaproteobacteria, were ignored. The 25 most abundant taxa accounted for 58% of the total sequence reads, and are listed in order of decreasing abundance in Figure 3. Family-level composition differed among assemblages, with inverse relationships in abundance of particular taxa in assemblages A and C. Families that were relatively abundant in assemblage A also tended to be abundant in assemblage B, whereas assemblages C and D also showed similar trends. The families Nitrosopumilaceae, Woeseiaceae, NB1-j, Actinomarinales, Kiloniellaceeae, Subgroup 22, OM190, and AT-s2-59 were particularly abundant in assemblage A. Haliaceae, BD7-8, and Methyloligellaceae were most prominent in assemblage B. In assemblage C, Flavobacteraceae, Rhodobacteraceae, Rubritalaceae, and Psychromonadaceae were more abundant. Gammaproteobacteria *Incertae sedis*, Desulfobulbaceae, Sva1033, Anaerolineaceae, and Desulfobacteraceae were abundant in assemblage D.

Indicator taxa analyses revealed that assemblage A had the highest number of indicator OTUs (294), followed by C (61) and D (27), and no OTUs were identified as indicators for assemblage B (Supplementary Figure 2 and Supplementary Table 1). Taxa indicative of a combination of assemblages will be referred to as indicative taxa, to differentiate from assemblage-specific indicator taxa. Mirroring trends shown in Figure 3, assemblages A + B and C + D shared relatively high numbers of indicative OTUs. No indicative taxa were shared between assemblages A + C alone, but some were shared between A + B + C, suggesting B represents a transitional community with representatives from both of the distinct A and C assemblages. Assemblages B + D also shared very few indicative taxa, but more were found when combined with either assemblage A or C (A + B + D or B + C + D).

**FIGURE 4:**
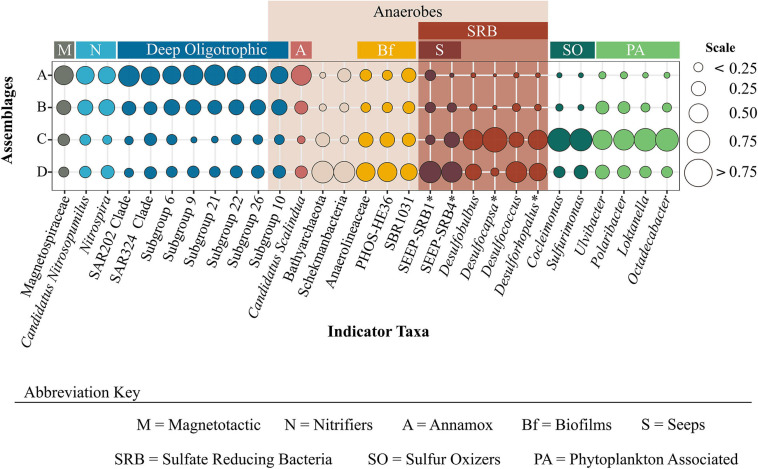
Proportion of assemblage-specific indicator taxa across prokaryotic assemblages. Indicator taxa are identified to the lowest taxonomic level possible. The plot is colored by functional groups which provide insight into characteristics associated with specific assemblages. *Indicates those taxa that were not yielded as indicators, but represent OTUs belonging to the same family as unidentified indicator taxa.

In light of the numerous uncultured and indicative OTUs, we limit our discussion to selected assemblage-specific indicator taxa, identified to the highest taxonomic resolution possible, for which published information could be used with confidence to infer function or distribution of a specific assemblage, and/or metabolic type or relationship to environmental variables (Figure 4). Indicator OTUs for assemblage A belonged to the class Acidobacteria (Subgroups 6, 9, 10, 21, 22, and 26), clades SAR202 and 324, and the families Thermoanaerobaculaceae, Magnetospiraceae (*Magnetospira*), Nitrosopumilaceae (*Candidatus Nitrosopumilus*), Nitrospiraceae (*Nitrospira*), and Scalinduaceae (*Scalindua*). Representative taxa under Thermoanaerobaculaceae belong to Acidobacteria subgroup 10 which was recently assigned to this family (Dedysh and Yilmaz, 2018). Indicator taxa for assemblage C included representatives from the families Flavobacteriaceae (*Polaribacter* and *Ulvibacter*), Rhodobactereacae (*Loktanella* and *Octadecabacter*), Thiovulaceae (*Cocleimonas*), Thiotrichaceae (*Sulfurimonas*), and Desulfobulbaceae (*Desulfobulbus*) (Figure 4). Assemblage D indicator taxa were within the families Anaerolineaceae, Desulfobacteraceae, Desulfobulbaceae, orders PHOS-HE36 and SBR1031, and phyla Bathyarchaeota, and Schekmanbacteria. The only indicator OTU identified down to genus level here was *Desulfococcus* (Desulfobacteraceae), therefore other abundant genus-level OTUs that belonged to the families Desulfobacteraceae and Desulfobulbaceae, were also queried and further explored with the indicator taxa (Figure 4).

### Environmental Correlates of Community Structure

**FIGURE 5:**
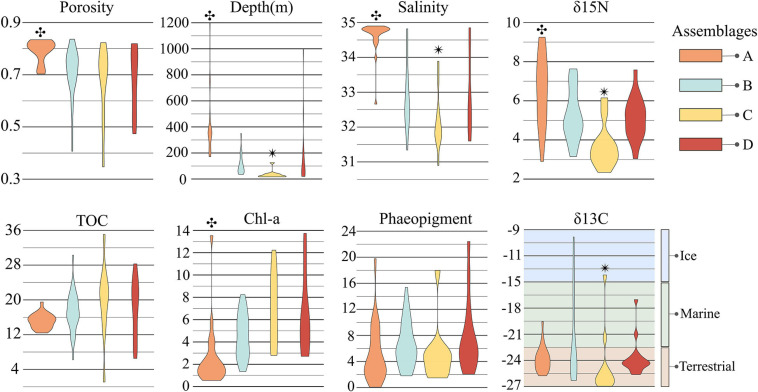
Distribution of environmental parameters for each assemblage. Each violin plot shows the distribution, within each assemblage, of the environmental parameters indicated as significant correlates in the ideal model for CAP analysis. Note that salinity is also included in this figure, though it was not run in CAP analysis due to the covariance inflation factor with depth. The symbols 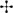 and 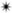 indicate significant differences between un-matching symbols with other assemblages. δ^13^C values for ice, marine, and terrestrial OM sources was derived from Dunton et al. (2006).

**FIGURE 6:**
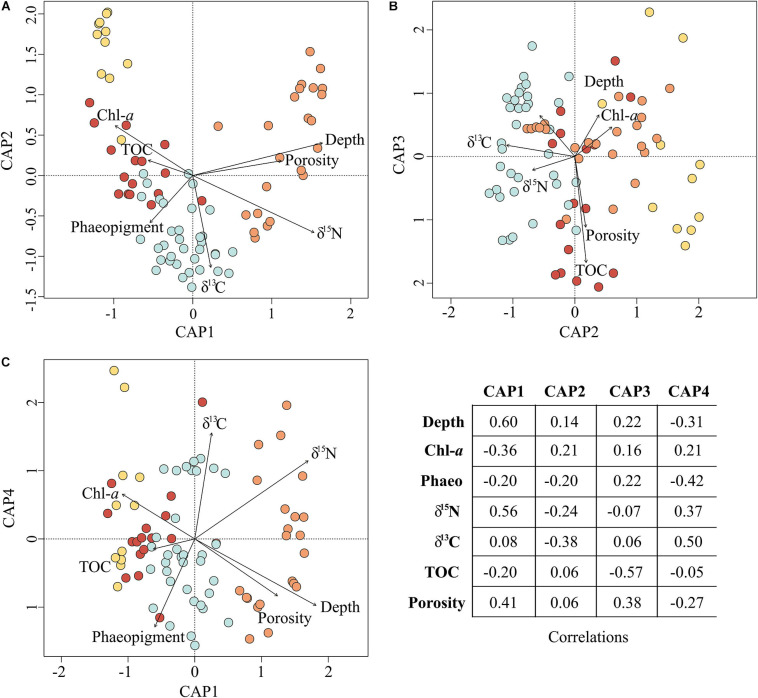
Environmental parameters influencing prokaryotic assemblage structure. The ordination plots above depict the results of Constrained Analysis of Principal Coordinates. All included environmental parameters are significant correlates (*P* < 0.001) to the first four CAP axes **(A–C)** and, in total, explained 18% of the variation in prokaryotic community structure (*R*^2^ = 0.18). The corresponding correlations are reported in the table for all axes.

Overall, the suite of environmental variables measured here explained relatively little (18%; Adj. *R*^2^ = 0.18) of the variation in prokaryote community structure among sites (Figure 6). Nonetheless, results of the CAP analysis did suggest significant correlations (adonis: *P* < 0.001) with different habitat characteristics for each assemblage. Assemblage A was found solely on the continental slope in 23 samples ranging from 171 to 1,200 m water depth (median depth 350 m), thus generally consisting of “deep-sea” locations further offshore and below the photic zone. Sediments at these locations exhibited significantly higher δ^15^N and porosity, and lower values of Chl-*a* concentration and C:N; bottom waters were warmer and more saline (Figure 5). Assemblage A largely differentiated from the other assemblages on the CAP1 axis (Figures 6A,C). CAP1 was most strongly and positively correlated with depth, δ^15^N, and porosity and exhibited a moderate to weak negative correlation with Chl-*a* and TOC. Variation within assemblage A was most apparent on the CAP4 axis suggesting Amundsen Gulf and Banks Island samples (+CAP4) are more influenced by degraded and marine derived OM (higher δ^15^N and δ^13^C) whereas Beaufort slope samples (-CAP4) had higher porosity sediments with comparatively more phaeopigment and terrestrial OM (Figure 6C).

Assemblage B consisted of 35 samples ranging between 36 and 350 m water depth (median depth 75 m), with a little more than half (19) located on the shelf and the remainder (16) on the slope. Median values of all environmental parameters for assemblage B were not significantly different than those for any of the other assemblages (Figure 5). CAP analysis showed a separation of assemblage B largely along the CAP2, axis suggesting that stations in this assemblage are characterized by more degraded OM (positive correlation with phaeopigment and δ^15^N) and less influence from terrestrial organic matter (higher δ^13^C suggesting marine or ice-algal source; Dunton et al., 2006; Figures 5, 6A). Differences among samples within assemblage B were most apparent on both the CAP3 and CAP4 axes, indicating that Amundsen Gulf and Banks Island samples (-CAP3) were generally characterized by higher TOC and lower porosity, whereas samples from the open shelf and slope (+CAP3) were characterized by higher porosity sediments with comparatively less TOC and more degraded phytodetritus (higher phaeopigment). Easternmost samples from the Amundsen Gulf (+CAP4) were separated from all other samples (-CAP4), with potentially sea-ice derived OM (higher δ^13^C), compared to more degraded marine and terrestrial OM (lower δ^13^C and higher phaeopigment) in the western Beaufort. Depth was also moderately or highly correlated with the CAP 3 and 4 axes, although this may reflect effects of other environmental variables that co-vary with depth.

The 11 samples represented by assemblage C were located solely in shallow shelf sediments ranging from 17 to 42 m water depth (median depth 21 m) with significantly lower values of δ^15^N, δ^13^C, and salinity. Assemblages C, B, and D were largely separated along the CAP2 axis, with assemblage C samples more correlated with fresh phytodetritus (higher Chl-a, lower δ^15^N) and terrestrial OM (lower δ^13^C). Samples within assemblage C varied along CAP3 axis which is most strongly correlated with TOC and porosity (Figure 6B). Nearshore samples from the Alaskan Beaufort shelf (+CAP3) were characterized by lower TOC and higher porosity, whereas those from the Canadian Beaufort and Amundsen Gulf (-CAP3) had higher TOC and lower porosity.

Assemblage D consisted of 16 samples which were collected almost exclusively at shelf locations (=100 m; median depth 72 m), except for 3 deep sites (=200 m) offshore of the Colville River plume where high concentrations of Chl-*a* were found. Environmental characterization for and correlations with assemblage D were not well captured with parameters measured here (Figures 5, 6). Variation within assemblage D was most apparent on the CAP3 axis, which suggests that several samples had comparatively higher organic matter input and lower porosity.

### Broader Geographical Context

**FIGURE 7:**
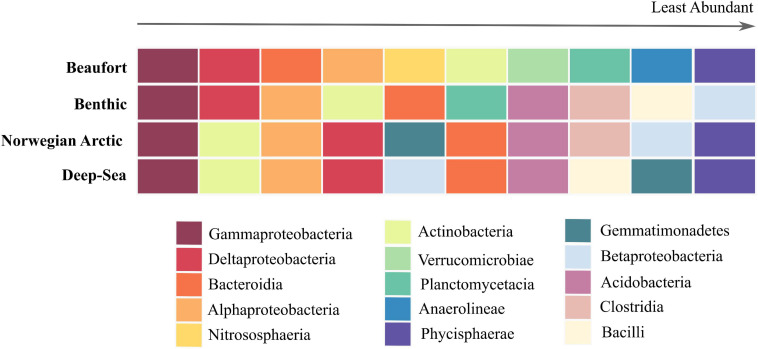
Top 10 abundant prokaryotes at class level exhibited in marine sediments. The colored grid above is arranged with surface sediment microbiomes on the *y*-axis, Beaufort, global benthic, Norwegian Arctic, and deep-sea (Zinger et al., 2011; Bienhold et al., 2016). The *x*-axis represents the rank from the most abundant taxa on the left to the least abundant taxa on the right exhibited by these microbiomes. The colors represent the class-level taxonomy of these prokaryotes.

Comparisons of the top 10 class-level taxa between Beaufort Sea, global benthic, deep-sea, and Norwegian-Arctic surface sediments indicate that all four sediment microbiomes share five major taxa: Gammaproteobacteria, Alphaproteobacteria, Deltaproteobacteria, Bacteroidia, and Actinobacteria (Figure 7). The Beaufort Sea sediment microbiome included taxa that were also highly abundant in either the combination of global benthic and deep-sea microbiomes (Plantomycetia) or the Norwegian-Arctic microbiome (Phycisphaera). Interestingly, four classes prevalent in the other three microbiomes were not among the 10 most abundant in Beaufort Sea sediments: Clostridia, Betaproteobacteria, Bacilli, and Acidobacteria. Nonetheless, Acidobacteria was quite abundant in the Beaufort Sea, falling within the top 13 most abundant classes. Class Betaproteobacteria was not detected in Beaufort Sea surface sediments, whereas Clostridia and Bacilli were present but in relatively low abundances. Nitrososphearia, Verrucomicrobia, and Anaeronlinae were among the top 10 taxa of the Beaufort Sea sediment microbiome, but were not abundant in the other datasets.

## Discussion

We identified four distinct assemblages, A, B, C, and D, across a broad area in southern Beaufort Sea surface sediments. Assemblage A occurred only at sites on the upper continental slope with lower concentrations of more degraded OM and higher porosity sediments. A distinct community (assemblage C) was found in shallow shelf sediments, with higher OM content and terrestrial OM influence. The wide distribution of assemblage B across shelf and slope depths and overlap in taxonomic composition with assemblages A and C, may reflect a transition zone between the shelf and slope sites containing generalists found in both bathymetric zones. Assemblage D was found primarily at shelf depths, but included a few deeper slope sites in the westernmost part of the study area with high OM content. Indicator taxa with known metabolic functions and/or ecological context suggest that assemblage D was not merely comprised of generalists. In order to provide ecological context and better understand what distinguishes these assemblages, we examined taxa that were assemblage-specific and/or exhibited higher abundances associated to an individual assemblage together with environmental correlates.

### Upper Slope Assemblage (A)

The indicator taxa of assemblage A, specifically Acidobacteria subgroups and SAR clades, reflect the deeper sampling depths (171–1,200 m) typified by reduced inputs of more degraded organic matter (Figure 5). Representatives of the phylum Acidobacteria found in Arctic deep-sea sediments have been proposed as potential indicators of oligotrophic conditions, and found to be more abundant in highly permeable sub-Arctic and Arctic sediments indicating an affinity for low-molecular-weight OM; subgroups 6, 10, and 26 in particular were more active under oligotrophic conditions (Bienhold et al., 2012; Hoffmann et al., 2017; Probandt et al., 2017). Here, Acidobacteria subgroups 6, 9, 10, 21, 22, and 26 along with SAR clades 324 and 202, were prevalent in slope assemblage A. The clades SAR324 and SAR202, like the Acidobacteria subgroups, are also indicative of deep, oligotrophic environments (Morris et al., 2004; Galand et al., 2010; Orcutt et al., 2011; Li et al., 2015; Landry et al., 2017; Nunoura et al., 2018; Zinke et al., 2018).

Slope assemblage A may also reflect some of the unique biogeochemical characteristics on the Beaufort Sea slope. In the Arctic Halocline waters over the Canada Basin, SAR 202 have been implicated in terrestrial OM degradation, consistent with the relatively strong terrestrial signal exhibited throughout the Southern Beaufort, even in the upper slope sediments (Figure 5; Colatriano et al., 2018). SAR202 has also been linked with deep-sea mud volcanoes and SAR324 with methane-rich hydrothermal plumes (Sheik et al., 2014; Coelho et al., 2016). Acidobacteria subgroup 10 was recently placed in the family Themoanaerobaculaceae which encompasses thermophilic obligate anaerobes isolated from marine hydrothermal vents and freshwater hot springs (Losey et al., 2013; Dedysh and Yilmaz, 2018). Several mud volcanoes on the Beaufort Sea slope release subsurface methane and produce temperatures ≥23°C within a few centimeters of the sediment-water interface, creating suitable habitat for thermophilic anaerobes such as those within subgroup 10 (Paull et al., 2015; Gamboa et al., 2017; Lee et al., 2018; Paull, pers. comm.). Subgroup 10 is widespread across the study area, yet to date, mud volcanoes have only been reported on the Canadian Beaufort Slope. These microbes may be spore-forming, similar to other thermophilic anaerobes reported from Arctic marine sediments that produce spores that lie dormant until favorable conditions arise (Hubert et al., 2010; Müller et al., 2014). However, previous reports of thermo-anaerobic bacterial spores belonged to the phylum Firmicutes, and not Acidobacteria, and none of the current taxa within Acidobacteria subgroup 10 are known to be spore-forming (Hubert et al., 2010; Losey et al., 2013; Müller et al., 2014). Given their occurrence in Arctic sediments from other regions, it is more likely that bacteria in subgroup 10 inhabit a wider temperature range than previously recognized (Hoffmann et al., 2017).

Beaufort sediment mineralogical properties may be influenced by the abundant indicator OTUs within Magnetospiraceae, a family composed of magnetotactic bacteria. Magnetotactic bacteria are unique in that they biosynthesize a mineral nanocrystal which they can use as a “compass” to navigate toward favorable redox conditions found at the oxic-anoxic interface in marine sediments (Lefevre and Bazylinski, 2013). Though indicator OTUs were all uncultured, *Magnetospira* is the most abundant taxon identified to genus-level within this family. Members of the genus *Magnetospira* produce magnetite crystals, which are the predominant magnetic grain found in Canadian Beaufort sediments and are more prevalent on the slope (Williams et al., 2012; Lefevre and Bazylinski, 2013; Gamboa et al., 2017).

Genera tightly linked to nitrification and anaerobic ammonia oxidation were relatively more abundant in slope assemblage A. *Nitrospira* are exclusively chemoautotrophic nitrite-oxidizing bacteria, oxidizing nitrite to nitrate, but have recently been found to perform complete nitrification, oxidizing ammonia to nitrate (Daims, 2014; Daims et al., 2015). *Candidatus Nitrosopumilus* encompasses ammonia-oxidizing archaea (AOA), which are aerobic nitrifiers that exhibit an extremely high affinity for ammonia, and can oxidize ammonia to nitrate, nitrite, or nitrous oxide (Stahl and de la Torre, 2012; Könneke et al., 2014; Martens-Habbena et al., 2015; Qin et al., 2016). AOA can perform mixotrophy and heterotrophy, but are largely chemoautotrophs (Stahl and de la Torre, 2012; Offre et al., 2013; Könneke et al., 2014; Qin et al., 2014). They also exhibit photoinhibition and increased autotrophic activity, which is consistent with the distribution of this assemblage at deeper sampling stations (Alonso-Sáez et al., 2012; Qin et al., 2014). In the pelagic zone of the Beaufort Sea, AOA are relatively more abundant in bottom waters on the slope and may be capable of urea degradation to fuel nitrification, as has been observed for *Candidatus Nitrosopumilus sediminis* in Arctic sediments near Svalbard (Alonso-Sáez et al., 2012; Park et al., 2012; Damashek et al., 2017). In the portion of our study area characterized by assemblage A, a peak in urea concentration occurs at 250–300 m water depth due to zooplankton and fish excretion, which may fuel the activity of AOA in this region (Simpson et al., 2008).

The genus *Candidatus Scalindua* encompasses anaerobic ammonia oxidizing (anammox) bacteria which convert ammonia to N_2_ via autotrophic metabolism and can utilize nitrite, nitrate, metal oxides, and even oligopeptides and small organic molecules as electron acceptors (Penton et al., 2006; Van de Vossenberg et al., 2013). Anaerobic ammonia oxidation may be a major sink for nitrate in the marine system, and has been reported in Arctic shelf slope sediments in the Chukchi Sea, near Greenland, and in deep-sea sediments from the Arctic mid-ocean ridge (Rysgaard et al., 2004; Penton et al., 2006; Jorgensen et al., 2012). The predominance of anammox bacteria at the deeper slope sites occupied by assemblage A supports previous claims that anammox is more likely to occur in areas with less organic matter loading (Thamdrup and Dalsgaard, 2002; Rysgaard et al., 2004; Burgin and Hamilton, 2007; McTigue et al., 2016).

### Generalist Assemblage (B)

While no indicator taxa were identified for assemblage B, one abundant taxon included members of the bacterial family Methyloligellaceae, dominated in this study by the genus *Methyloceanibacter* which contains known methylotrophs (Takeuchi et al., 2014; Vekeman et al., 2016a). Some species of *Methyloceanibacter* oxidize methanol, while others exhibit methane oxidation and include the methane monooxygenase gene in their genome (Takeuchi et al., 2014; Vekeman et al., 2016a,b). We have detected a relatively high abundance of the methane monooxygenase gene in these sediments through metagenomics analyses, although further analyses are needed to attribute these genes to a specific taxonomic group (Walker et al. unpublished data). Nonetheless, the presence of *Methyloceanibacter* coupled with the abundant distribution of the methane monooxygenase gene in methane-permeated Beaufort Sea sediments suggests that aerobic methane oxidation may be an active process in this region, although further research confirming this activity would be required. As there are multiple sources of methane in these sediments, methane oxidation in the Beaufort Sea is likely an important process constraining methane concentrations reaching the sea surface (Lorenson et al., 2016). However, no evidence for methanotrophy has yet been reported in the water column or subsurface sediments, despite chemical evidence that oxidation of methane derived from ancient sources may be occurring near the sediment-water interface (Hamdan et al., 2013; Sparrow et al., 2018).

### Shallow Shelf Assemblage (C)

Indicator taxa for assemblage C were characteristic of a shallow-shelf environment influenced by fresh phytodetritus, riverine input, and sea ice, in keeping with elevated Chl-a and significantly lower values of δ^13^C and salinity observed at assemblage C locations. Many of these taxa may be deposited to the seafloor in association with sinking particles. Flavobacteriaceae occur in conjunction with phytoplankton blooms and subsequent deposition of phytodetritus (Junge et al., 2002; Bienhold et al., 2012; Teeling et al., 2012; Hoffmann et al., 2017). Genera within this family are typically aerobic, though there are some facultative anaerobic heterotrophs capable of degrading complex polysaccharides (Teeling et al., 2012; McBride, 2014; Xing et al., 2015). Two of the genus-level indicator taxa found here, *Ulvibacter and Polaribacter*, are often particle attached and tightly connected with phytoplankton blooms and sea ice (Junge et al., 2002; Bowman and McCuaig, 2003; Brinkmeyer et al., 2003; Teeling et al., 2012; Xing et al., 2015). *Ulvibacter* is one of the first and most abundant genera to respond to a phytoplankton bloom following algal lysis, whereas *Polaribacter* is most abundant in later stages of an algal bloom and can use sulfatases to access highly sulfated algal material (Teeling et al., 2012, 2016; Xing et al., 2015; Helbert, 2017). *Polaribacter* species generally include psychrophilic bacteria that are most commonly found in polar and temperate regions. Until recently, they had only been identified in sea ice and seawater, but are now described from Arctic sediments offshore of Svalbard (Gosink et al., 1998; Bowman et al., 2012; Li et al., 2014; Rapp et al., 2018).

A few of the indicator taxa for assemblage C suggest that light availability may be a major factor influencing community composition. Genomic analyses of *Polaribacter* species, along with *Loktanella* and *Octadecabacter*, indicator taxa from the Rhodobacteraceae family, contain various rhodopsins (light-driven proton pumps) and other light responsive genes, indicating an affinity for well-lit shallower waters (Gonzalez et al., 2008; Boeuf et al., 2013; Vollmers et al., 2013; Wu et al., 2014; Xing et al., 2015; Dogs et al., 2017). *Loktanella*, described as anoxygenic photoheterotrophs (AAP), have been found in Beaufort Sea surface waters including the Mackenzie River outflow where they were positively correlated with Chl-*a* (Boeuf et al., 2013). AAP photosynthesize but also require oxygen for breaking down organic matter with light-harvested energy, and are most abundant in coastal waters and eutrophic estuaries (Waidner and Kirchman, 2007; Boeuf et al., 2013). Photoheterotrophy could be an advantage in such environments that are heavily influenced by terrestrial material, providing an energy source to fuel more costly metabolic processes such as the degradation of humic substances (Boeuf et al., 2013). *Octadecabacter* species are psychrophilic, aerobic heterotrophs with gas vesicles and are abundant in sea ice, though a few taxa have been isolated from seawater and marine sediments (Gosink et al., 1997; Brinkmeyer et al., 2003; Vollmers et al., 2013; Lee et al., 2014; Billerbeck et al., 2015). *Octadecabacter* spp. have also been linked to phytoplankton blooms and detrital aggregates in the Arctic and elsewhere (Grossart et al., 2005; Rapp et al., 2018; Koedooder et al., 2019).

The remaining indicator taxa for assemblage C belong to the bacterial families Thiotrichaceae, Thiovulaceae, and Desulfobulbaceae, all of which are involved in the marine sulfur cycle. Members of the Desulfobulbaceae family, which are anaerobic sulfate reducers, were also found in assemblage D and discussed further below (Kuever, 2014b). Thiotrichaceae and Thiovulaceae, represented here by *Cocleimonas* and *Sulfurimonas*, respectively, are sulfur oxidizers (Tanaka et al., 2011; Garrity et al., 2015; Han and Perner, 2015). Sulfur oxidizing bacteria are generally limited by the availability of necessary electron donors (typically reduced sulfur compounds) and acceptors (oxygen, nitrate) favored in anoxic and oxic conditions, respectively (Wasmund et al., 2017; Jørgensen et al., 2019). Adaptations exhibited by *Sulfurimonas* and *Cocleimonas* provide a competitive advantage in the oxidation of elemental sulfur and other sulfur intermediates (Tanaka et al., 2011; Pjevac et al., 2014; Han and Perner, 2015; Wasmund et al., 2017).

### Anoxic Assemblage (D)

The indicator taxa for assemblage D suggest a greater extent of anoxic sediment within the upper 1 cm layer. All indicator OTUs for Assemblage D are strict anaerobes within the families Desulfobulbaceae, Desulfobacteraceae, Anaerolineaceae, PHOS-HE36, the order SBR1033, and the phyla Bathyarcheaota and Schekmanbacteria (Kuever, 2014a,b; Evans et al., 2015; Anantharaman et al., 2018; Yamada and Sekiguchi, 2018). Members of the archaeal phylum Bathyarcheaota and candidate bacterial phylum Schekmanbacteria consisted of uncultured OTUs with less than 85% matches to published sequences in BLAST. Though inferences based on this level of taxonomy are highly speculative, representatives from both phyla have been most commonly sequenced from subsurface marine sediments (Inagaki et al., 2003; Biddle et al., 2006; Sørensen and Teske, 2006; Evans et al., 2015; Anantharaman et al., 2018; Lopez-Fernandez et al., 2018; Winkel et al., 2018).

Anaerolineaceae are comprised of anaerobic heterotrophs with a fermentative metabolism and have been sequenced in anoxic, organic-rich habitats such as sludge digesters, hydrothermal vents, and glacial fjord sediments off the coast of Svalbard (McIlroy et al., 2017; Zeng et al., 2017; Yamada and Sekiguchi, 2018). They are typically abundant in biofilms and implicated as hydrogenotrophs and alkane degraders, though few studies have examined marine representatives (Siegert et al., 2011; Sinkko et al., 2013; Al-Thani et al., 2014; Liang et al., 2015; van der Waals et al., 2017). Similarly, bacteria in the order SBR1031 have been primarily investigated in waste-water treatment plants where they produce biofilms (Xia et al., 2016; Wang X. et al., 2018). Marine representatives have been found in oil-contaminated estuarine sediments, and increase in abundance in seasonal microbial mats in the North Sea (Obi et al., 2017; Cardoso et al., 2019). Bacteria from the family PHOS-HE36 have also been predominately studied in the context of sludge digesters and biofilms, often sequenced with bacteria in the Anaerolineaceae family, but have also been found in marine sediments near methane seeps, hydrothermal vents, and within microbial mats (Dabert et al., 2001; Fujii et al., 2002; Trembath-Reichert et al., 2016; Wang L. et al., 2018; Zinke et al., 2018; Yang et al., 2019).

Indicator taxa from assemblage D are also connected to the sulfur cycle, specifically sulfate reduction, which is the dominant process responsible for anaerobic degradation of organic matter in marine sediments (Wasmund et al., 2017; Jørgensen et al., 2019). The abundance of several taxa within the families Desulfobacteraceae and Desulfobulbaceae suggest that sulfate reduction is a prevalent in both shallow shelf assemblage C and anoxic assemblage D. At family level, Desulfobacteraceae taxa were more abundant in assemblage D, whereas Desulfobulbaceae taxa were more evenly distributed between assemblages C and D, albeit with specific genera that were more abundant in shallow assemblage C. These families have shown responses to different substrates in Arctic sulfidic sediments, suggesting the taxonomic differences between assemblages C and D reflect differences in substrate availability among sites (Dyksma et al., 2018; Müller et al., 2018).

*Desulfocapsa* and *Desulfobulbus* (Desulfobulbaceae) were more abundant in shallow shelf assemblage C, and have often been sequenced in Arctic fjord sediments alongside sulfur-oxidizing bacteria (SOB) such as *Sulfurimonas* and *Cocleimonas;* which were also identified as indicator taxa for assemblage C (Zeng et al., 2017; Trivedi et al., 2018; Buongiorno et al., 2019). This repeated co-occurrence in Arctic marine sediments suggests an affinity for similar environmental/redox conditions, and further exemplifies subtle differences between sulfur cycling in assemblages C and D.

The more abundant SRB taxa in assemblage D, particularly SEEP-SRB1 and SEEP-SRB4 clades, are most commonly sequenced from cold seeps (Schreiber et al., 2010; Kleindienst et al., 2012; Ruff et al., 2015). SEEP-SRB have been implicated in coupling sulfate reduction to the anaerobic oxidation of non-methane and methane hydrocarbons, however they remain largely uncultured (Petro et al., 2019). *Desulfococcus* spp., though commonly found in sulfidic sediments, are also associated with marine seeps, and may be major players in anaerobic hydrocarbon degradation in association with seeps (Ravenschlag et al., 2000; Orphan et al., 2001; Kleindienst et al., 2012, 2014).

The majority of samples in this assemblage were located in shallow shelf sediments, some of which were characterized by heavy OM loading and low porosity, conducive to oxygen depletion. Samples collected on the slope in assemblage D exhibited some of the highest concentrations of Chl-*a* measured here, and may have received a recent pulse of fresh phytodetritus leading to shallow anoxic conditions in sediments. Moreover, multiple indicator taxa for assemblage D are also associated with biofilms, microbial mats, seep environments, or subsurface sediments, suggesting the presence of these features at some sites. Microbial mats have been reported on Beaufort Sea sediments, mostly surrounding slow gas seeps (Paull et al., 2011). The inherently patchy distribution of benthic seeps and microbial mats coupled with the varying conditions that foster anoxic sediments may explain the lack of definable substructure exhibited by assemblage D and weak correlations with environmental parameters in this study.

### Broader Geographical Context

Benthic prokaryotic community substructure on the Beaufort Sea shelf and upper slope broadly reflected longitudinal gradients in OM quality and quantity, as well as the influence of bathymetry. While bathymetric effects were particularly apparent in distinguishing slope assemblage A, shallow shelf assemblage C, and generalist assemblage B, redox conditions likely played a role in differentiating assemblages C and D. These findings highlight the importance of assessing prokaryotic communities on a broad spatial scale, given that community structure reflected environmental variation occurring over scales of meters to kilometers. This raises the question of how prokaryotic community structure in the Beaufort Sea compares to other regions within the Arctic and beyond.

At class-level, 60% of the top 10 taxa from Beaufort Sea sediments were shared with those from the global, deep-sea, and Norwegian Arctic sediment microbiomes (Figure 7). The classes Anaerolineae, Nitrososphearia, and Verrucomicrobiae were abundant and unique to the Beaufort Sea surface sediment microbiome. The bacterial class Anaerolineae and archaeal class Nitrososphearia are typified by the families Anaerolinaceae and Nitrosopumilaceae which, as mentioned above, primarily contain anaerobic bacteria associated with heavy OM loading as was prevalent in anoxic assemblage D, and ammonia-oxidizing archaea prevalent in slope assemblage A, respectively. Not surprisingly, Nitrososphearia was not among the most abundant taxa in the global, deep-sea, or Norwegian Arctic sediment microbiomes. Though common in marine surface sediments and typically more abundant than ammonia oxidizing bacteria, archaea have been overlooked in previous studies solely focused on bacteria (Park et al., 2010; Zinger et al., 2011; Bienhold et al., 2016). Their relatively high abundance here highlights the under-appreciated role of archaea in marine surface sediments. If archaea are excluded, the bacterial taxon Thermoanaerobaculia joins the top 10 most abundant taxa in our study. This group encompasses the anaerobic, thermophilic organisms largely represented by subgroup 10 an indicator taxon for slope assemblage A. This class was not among the most abundant taxa in sediments globally, in the deep sea, or in the Norwegian Arctic. Thermoanaerobaculia together with Anaerolineae suggest comparatively shallower anoxic conditions in Beaufort Sea surface sediments, spanning both slope and shallow shelf locations.

Betaproteobacteria, Gemmatimonadetes, Bacilli, and Clostridia were among the 10 most abundant taxa from global benthic, deep-sea, and Norwegian-Arctic microbiomes, yet were not prominent in Beaufort sediments. Betaproteobacteria, which were not present even at low abundances in Beaufort Sea sediments, were discovered to be potential laboratory contaminants in other studies (Kulakov et al., 2002; Grahn et al., 2003; Laurence et al., 2014; Salter et al., 2014). On a global scale Zinger et al. (2011) observed Bacilli and Clostridia in higher abundances in coastal sediments vs. open water or deep-sea sediments. Bacilli and Clostridia can be indicators of urban fecal contamination in coastal watersheds, lagoons, and nearshore seawater (Wu et al., 2010; Dubinsky et al., 2012; Dai et al., 2018). As the Beaufort Sea is extremely remote from urban life, it is not surprising that these taxa were less prevalent. Clostridia was included in the Norwegian-Arctic microbiome and Bacilli in the deep-sea microbiome, which are also presumably more pristine than typical coastal areas, suggesting that there are bacteria in these classes which are not necessarily correlated with urban waste (Bienhold et al., 2016).

The prokaryotic community structure in the Beaufort Sea differed somewhat from the Norwegian-Arctic, even at the relatively coarse taxonomic level of class, suggesting benthic microbes may reflect regional differences in hydrography, biogeochemistry, and bathymetry of Arctic shelf systems (Carmack and Wassmann, 2006). However, samples from the Norwegian-Arctic were largely collected at depths > 1,000 m, so these regional differences need to be further explored particularly across wider depth ranges.

### Synthesis

The occurrence of strictly anaerobic taxa in all assemblages identified here indicates that Beaufort Sea surface sediments exhibit widespread anoxia within the 0–1 cm depth horizon, consistent with oxygen penetration studies conducted in other Arctic sediments (Kostka et al., 1999; Jørgensen et al., 2005). A larger proportion of anaerobic taxa occurred in shallow shelf (C) and anoxic (D) assemblages, indicating that, in general, oxygen depletion in nearshore sediments is comparatively more widespread than offshore, particularly in areas where organic matter loading and Chl-*a* were higher and porosity lower. Although this is a common trend when comparing shelf to slope sediments, it may be of particular importance in the Beaufort Sea when viewed through the lens of climate change (Orcutt et al., 2011). An overall net increase of river runoff and terrestrial organic matter has already been established for the Mackenzie River, and is predicted to continue over the coming years (Holmes et al., 2012; Doxaran et al., 2015). Thus, we might expect an increase in abundance of anaerobic taxa in shallow shelf assemblage C, with potentially more stations resembling or overlapping with anoxic assemblage D. There may also be increased taxonomic overlap between microbial communities associated with the Mackenzie River outflow and shallow shelf assemblage C. In addition to riverine input, shifts in primary production and sea-ice extent will likely be most apparent in the shallow shelf sediments, as the majority of indicator taxa in assemblage C have been strongly correlated to both phytoplankton blooms and sea-ice microbial communities.

An increase in OM input, whether terrestrial or marine derived, will likely translate to an increase in OM burial and subsequent methanogenesis in the already methane-permeated Beaufort Sea sediments (Coffin et al., 2013). Characterizing the microbes involved in both methane production/degradation and associated processes in these sediments is thus of particular interest. The high relative abundance of potential methanotrophs exhibited in this study could, in addition to highlighting the presence of methane throughout sediments in the region, indicate an important constraint on methane efflux that requires further investigation.

This study also highlights the vast array of uncultured and unclassified microbes in Arctic marine sediments, particularly exemplified by slope assemblage A, which was the most divergent and diverse and contained the highest number of uncultured taxa across all assemblages. Anoxic assemblage D also exhibited a comparatively high abundance of uncultured microbes with representatives which are more rarely studied in the context of the marine system. The lack of knowledge regarding these taxa points to a need for more experimental and culture-based studies to elucidate their role in biogeochemical processes, that would provide insights into the links between microbial community structure and benthic ecosystem function. Although taxonomic classification does not directly equate to function, known microbes from slope assemblage A highlight how taxonomic composition may provide a framework for future studies. For instance, research investigating the role of nitrification and anammox in the Beaufort Sea would be most beneficial in slope sediments where microbes involved in aerobic/anaerobic ammonia oxidation and nitrite oxidation were abundant, and could reveal useful information about regional nitrogen cycling and upwelling dynamics. Additionally, microbial taxonomy can provide insights into environmental conditions that are not typically measured with surveys of larger benthic epi- and infaunal organisms, such as sediment oxygen penetration and the presence of biofilms, microbial mats, and/or seeps seemingly reflected by the taxonomic composition of anoxic assemblage D. Overall, these insights provided by prokaryotic community structure emphasize the value of incorporating microbial surveys more broadly into field sampling programs in the North American Arctic and elsewhere. This study establishes a baseline for Beaufort Sea benthic microbes, however without long-term datasets we cannot assess how these communities will respond to and reflect shifts related to climate change or other disturbances such as energy resource exploration.

## Data Availability Statement

The datasets presented in this study can be found in online repositories. The names of the repository/repositories and accession number(s) can be found below: https://www.ncbi.nlm.nih.gov/, PRJNA451041.

## Author Contributions

AW was involved in field work for sample collection, conducted all sample processing and data analysis, and took a lead role in the interpretation of results and manuscript preparation. ML contributed funding, advised on data analysis and interpretation, and contributed to manuscript preparation. SM conducted field work to collect samples and environmental data, provided funding, and contributed to project planning, interpretation of results, and manuscript preparation. All authors contributed to the article and approved the submitted version.

## Conflict of Interest

The authors declare that the research was conducted in the absence of any commercial or financial relationships that could be construed as a potential conflict of interest.

## Footnotes

1 https://github.com/amwalker/Beaufort-benthos

## Supplementary Material

Supplementary Figure 1 Distribution of *Methyloceanibacter* at different levels of sequence removal. The pie chart above illustrates an example, using the genus *Methyloceanibacter*, of how relative abundances change with removal of singletons and doubletons compared to no removal (none). The difference in abundance is attributed to the removal of OTUs that would likely not have been removed if clustered at 97% similarity as they belong to the same genus. This is an important consideration when using amplicon sequence variants (ASVs), and software which automatically remove singletons and doubletons such as DADA2.

Supplementary Figure 2 Indicator OTUs for individual and combinations of prokaryotic assemblages. This Venn diagram illustrates the number of indicator OTUs in parentheses identified for individual assemblages and those shared in different combinations of assemblages.

Supplementary Figure 3 Proportion of top 10 most abundant taxa in Beaufort sediments at class level. This bubble plot illustrates the proportion of the 10 most abundant taxa at class level for Beaufort Sea surface sediments.

Supplementary Table 1 Indicator taxa for each prokaryotic assemblage. Taxa are arranged from most abundant starting at the top to least abundant toward the bottom. Those without a semi-colon indicate OTUs which could not be IDed past the taxonomic level provided.

## References

[B1] ACIA (2005). *Arctic Climate Impact Assessment. ACIA Overview Report.* Available online at: http://www.amap.no/documents/doc/arctic-arctic-climate-impact-assessment/796 (accessed March 4, 2020).

[B2] Alonso-Sáez L. Waller A. S. Mende D. R. Bakker K. Farnelid H. Yager P. L. (2012). Role for urea in nitrification by polar marine Archaea. *Proc. Natl. Acad. Sci. U.S.A.* 109 17989–17994. 10.1073/pnas.1201914109 23027926 PMC3497816

[B3] Al-Thani R. Al-Najjar M. A. A. Al-Raei A. M. Ferdelman T. Thang N. M. Al Shaikh I. (2014). Community structure and activity of a highly dynamic and nutrient-limited hypersaline microbial mat in Um Alhool Sabkha, Qatar. *PLoS One* 9:e0092405. 10.1371/journal.pone.0092405 24658360 PMC3962408

[B4] Anantharaman K. Hausmann B. Jungbluth S. P. Kantor R. S. Lavy A. Warren L. A. (2018). Expanded diversity of microbial groups that shape the dissimilatory sulfur cycle. *ISME J.* 12 1715–1728. 10.1038/s41396-018-0078-0 29467397 PMC6018805

[B5] Apprill A. McNally S. Parsons R. Weber L. (2015). Minor revision to V4 region SSU rRNA 806R gene primer greatly increases detection of SAR11 bacterioplankton. *Aquat. Microb. Ecol.* 75 129–137. 10.3354/ame01753

[B6] Arar E. J. Collins G. B. (1997). *Method 445.0: In Vitro Determination of Chlorophyll a and Pheophytin a in Marine and Freshwater Algae by Fluorescence.* Ohio: United States Environmental Protection Agency, Office of Research and Development, National Exposure Research Laboratory.

[B7] Arnosti C. (2008). Functional differences between Arctic seawater and sedimentary microbial communities: contrasts in microbial hydrolysis of complex substrates. *FEMS Microbiol. Ecol.* 66 343–351. 10.1111/j.1574-6941.2008.00587.x 18778275

[B8] Bell L. E. Bluhm B. A. Iken K. (2016). Influence of terrestrial organic matter in marine food webs of the Beaufort Sea shelf and slope. *Mar. Ecol. Prog. Ser.* 550 1–24. 10.3354/meps11725

[B9] Bennett R. H. Lambert D. N. (1971). Rapid and reliable technique for determining unit weight and porosity of deep-sea sediments. *Mar. Geol.* 11 201–207. 10.1016/0025-3227(71)90007-7

[B10] Biddle J. F. Lipp J. S. Lever M. A. Lloyd K. G. Sørensen K. B. Anderson R. (2006). Heterotrophic Archaea dominate sedimentary subsurface ecosystems off Peru. *Proc. Natl. Acad. Sci. U.S.A.* 103 3846–3851. 10.1073/pnas.0600035103 16505362 PMC1533785

[B11] Bienhold C. Boetius A. Ramette A. (2012). The energy–diversity relationship of complex bacterial communities in Arctic deep-sea sediments. *ISME J.* 6 724–732. 10.1038/ismej.2011.140 22071347 PMC3309351

[B12] Bienhold C. Zinger L. Boetius A. Ramette A. (2016). Diversity and biogeography of bathyal and abyssal Seafloor Bacteria. *PLoS One* 11:e0148016. 10.1371/journal.pone.0148016 26814838 PMC4731391

[B13] Bier R. L. Bernhardt E. S. Boot C. M. Graham E. B. Hall E. K. Lennon J. T. (2015). Linking microbial community structure and microbial processes: an empirical and conceptual overview. *FEMS Microbiol. Ecol.* 113:fiv113. 10.1093/femsec/fiv113 26371074

[B14] Billerbeck S. Orchard J. Tindall B. J. Giebel H.-A. Brinkhoff T. Simon M. (2015). Description of *Octadecabacter temperatus* Sp. Nov., isolated from the Southern North sea, emended descriptions of the genus octadecabacter and its species and reclassification of octadecabacter jejudonensisPark and Yoon 2014 as pseudooctadecabacter jejudon. *Int. J. Syst. Evol. Microbiol.* 65 1967–1974. 10.1099/ijs.0.000205 25816810

[B15] BOEM (2019). *Leasing and Plans | BOEM.* Available online at: https://www.boem.gov/Alaska-Leasing-and-Plans/ (accessed January 14, 2019).

[B16] Boetius A. Ravenschlag K. Schubert C. J. Rickert D. Widdel F. Gleseke A. (2000). A marine microbial consortium apparently mediating anaerobic oxidation methane. *Nature* 407 623–626. 10.1038/35036572 11034209

[B17] Boeuf D. Cottrell M. T. Kirchman D. L. Lebaron P. Jeanthon C. (2013). Summer community structure of aerobic anoxygenic phototrophic bacteria in the western Arctic Ocean. *FEMS Microbiol. Ecol.* 85 417–432. 10.1111/1574-6941.12130 23560623

[B18] Bowman J. P. McCuaig R. D. (2003). Biodiversity, community structural shifts, and biogeography of prokaryotes within Antarctic continental shelf sediment. *Appl. Environ. Microbiol.* 69 2463–2483. 10.1128/AEM.69.5.2463-2483.2003 12732511 PMC154503

[B19] Bowman J. S. Rasmussen S. Blom N. Deming J. W. Rysgaard S. Sicheritz-Ponten T. (2012). Microbial community structure of Arctic multiyear sea ice and surface seawater by 454 sequencing of the 16S RNA gene. *ISME J.* 6 11–20. 10.1038/ismej.2011.76 21716307 PMC3246233

[B20] Braeckman U. Janssen F. Lavik G. Elvert M. Marchant H. Buckner C. (2018). Carbon and nitrogen turnover in the Arctic deep sea: in situ benthic community response to diatom and coccolithophorid phytodetritus. *Biogeosciences* 15 6537–6557. 10.5194/bg-15-6537-2018

[B21] Brinkmeyer R. Knittel K. Jürgens J. Weyland H. Amann R. Helmke E. (2003). Diversity and structure of bacterial communities in arctic versus Antarctic Pack Ice. *Appl. Environ. Microbiol.* 69 6610–6619. 10.1128/AEM.69.11.6610-6619.2003 14602620 PMC262250

[B22] Brothers L. L. Herman B. M. Hart P. E. Ruppel C. D. (2016). Subsea ice-bearing permafrost on the U.S. Beaufort Margin: 1. Minimum seaward extent defined from multichannel seismic reflection data. *Geochem. Geophys. Geosyst.* 17 4354–4365. 10.1002/2016GC006584

[B23] Buongiorno J. Herbert L. C. Wehrmann L. M. Michaud A. B. Laufer K. Røy H. (2019). Complex microbial communities drive iron and sulfur cycling in arctic fjord sediments. *Appl. Environ. Microbiol.* 85:e00949-19. 10.1128/aem.00949-19 31076435 PMC6606867

[B24] Burgin A. J. Hamilton S. K. (2007). Have we overemphasized the role of denitrification in aquatic ecosystems? A review of nitrate removal pathways. *Front. Ecol. Environ.* 5 89–96.

[B25] Buttigieg P. L. Ramette A. (2015). Biogeographic patterns of bacterial microdiversity in Arctic deep-sea sediments (HAUSGARTEN, fram strait). *Front. Microbiol.* 6:660. 10.3389/fmicb.2014.00660 25601856 PMC4283448

[B26] Caporaso J. G. Lauber C. L. Walters W. A. Berg-Lyons D. Huntley J. Fierer N. (2012). Ultra-high-throughput microbial community analysis on the Illumina HiSeq and MiSeq platforms. *ISME J.* 6 1621–1624. 10.1038/ismej.2012.8 22402401 PMC3400413

[B27] Caporaso J. G. Lauber C. L. Walters W. A. Berg-Lyons D. Lozupone C. A. Turnbaugh P. J. (2011). Global patterns of 16S rRNA diversity at a depth of millions of sequences per sample. *Proc. Natl. Acad. Sci. U.S.A.* 108 4516–4522. 10.1073/pnas.1000080107 20534432 PMC3063599

[B28] Cardoso D. C. Cretoiu M. S. Stal L. J. Bolhuis H. (2019). Seasonal development of a coastal microbial mat. *Sci. Rep.* 9:9035. 10.1038/s41598-019-45490-8 31227767 PMC6588573

[B29] Carmack E. Wassmann P. (2006). Food webs and physical–biological coupling on pan-Arctic shelves: unifying concepts and comprehensive perspectives. *Prog. Oceanogr.* 71 446–477. 10.1016/j.pocean.2006.10.004

[B30] Carmack E. C. Macdonald R. W. (2002). Oceanography of the Canadian shelf of the beaufort sea: a setting for marine life. *Arctic* 55 29–45. 10.14430/arctic733

[B31] Casciotti K. L. Buchwald C. (2012). Insights on the marine microbial nitrogen cycle from isotopic approaches to nitrification. *Front. Microbiol.* 3:356. 10.3389/fmicb.2012.00356 23091468 PMC3469838

[B32] Cock P. J. A. Antao T. Chang J. T. Chapman B. A. Cox C. J. Dalke A. (2009). Biopython: freely available Python tools for computational molecular biology and bioinformatics. *Bioinformatics* 25 1422–1423. 10.1093/bioinformatics/btp163 19304878 PMC2682512

[B33] Coelho F. J. R. C. Louvado A. Domingues P. M. Cleary D. F. R. Ferreira M. Almeida A. (2016). Integrated analysis of bacterial and microeukaryotic communities from differentially active mud volcanoes in the Gulf of Cadiz. *Sci. Rep.* 6:35272. 10.1038/srep35272 27762306 PMC5071872

[B34] Coffin R. Smith J. Yoza B. Boyd T. Montgomery M. (2017). Spatial variation in sediment organic carbon distribution across the alaskan beaufort Sea Shelf. *Energies* 10:1265 10.3390/en10091265

[B35] Coffin R. B. Smith J. P. Plummer R. E. Yoza B. Larsen R. K. Millholland L. C. (2013). Spatial variation in shallow sediment methane sources and cycling on the Alaskan Beaufort Sea Shelf/Slope. *Mar. Pet. Geol.* 45 186–197. 10.1016/J.MARPETGEO.2013.05.002

[B36] Colatriano D. Tran P. Q. Guéguen C. Williams W. J. Lovejoy C. Walsh D. A. (2018). Genomic evidence for the degradation of terrestrial organic matter by pelagic Arctic Ocean Chloroflexi bacteria. *Commun. Biol.* 1:90. 10.1038/s42003-018-0086-7 30271971 PMC6123686

[B37] Dabert P. Sialve B. Delgenès J.-P. Moletta R. Godon J.-J. (2001). Characterisation of the microbial 16S rDNA diversity of an aerobic phosphorus-removal ecosystem and monitoring of its transition to nitrate respiration. *Appl. Microbiol. Biotechnol.* 55 500–509. 10.1007/s002530000529 11398934

[B38] Dai T. Zhang Y. Ning D. Su Z. Tang Y. Huang B. (2018). Dynamics of sediment microbial functional capacity and community interaction networks in an Urbanized Coastal Estuary. *Front. Microbiol.* 9:2731. 10.3389/fmicb.2018.02731 30487783 PMC6246683

[B39] Daims H. (2014). “The family nitrospiraceae,” in *The Prokaryotes*, eds Balows A. Trüper H.G. Dworkin M. Harder W. Schleifer K.-H. (Berlin: Springer), 733–749.

[B40] Daims H. Lebedeva E. V. Pjevac P. Han P. Herbold C. Albertsen M. (2015). Complete nitrification by Nitrospira bacteria. *Nature* 528 504–509. 10.1038/nature16461 26610024 PMC5152751

[B41] Damashek J. Pettie K. P. K. Brown Z. W. Z. Mills M. M. M. Arrigo K. R. K. Francis C. A. C. (2017). Regional patterns in ammonia-oxidizing communities throughout Chukchi Sea waters from the bering strait to the Beaufort Sea. *Aquat. Microb. Ecol.* 79 273–286. 10.3354/ame01834

[B42] De Cáceres M. Legendre P. (2009). Associations between species and groups of sites: indices and statistical inference. *Ecology* 90 3566–3574. 10.1890/08-1823.1 20120823

[B43] De Cáceres M. Legendre P. Moretti M. (2010). Improving indicator species analysis by combining groups of sites. *Oikos* 119 1674–1684. 10.1111/j.1600-0706.2010.18334.x

[B44] Dedysh S. N. Yilmaz P. (2018). Refining the taxonomic structure of the phylum Acidobacteria. *Int. J. Syst. Evol. Microbiol.* 68 3796–3806. 10.1099/ijsem.0.003062 30325293

[B45] Deming J. W. Baross J. A. (1993). “The early diagenesis of organic matter: bacterial activity,” in *Organic Geochemistry*, eds Murphy M. T. J. Eglinton G. (Berlin: Springer), 119–144.

[B46] Dogs M. Wemheuer B. Wolter L. Bergen N. Daniel R. Simon M. (2017). Rhodobacteraceae on the marine brown alga Fucus spiralis are abundant and show physiological adaptation to an epiphytic lifestyle. *Syst. Appl. Microbiol.* 40 370–382. 10.1016/j.syapm.2017.05.006 28641923

[B47] Doxaran D. Devred E. Babin M. (2015). A 50% increase in the amount of terrestrial particles delivered by the Mackenzie River into the Beaufort Sea (Canadian Arctic Ocean) over the last 10 years. *Biogeosci. Discuss.* 12 305–344. 10.5194/bgd-12-305-2015

[B48] Dubinsky E. A. Esmaili L. Hulls J. R. Cao Y. Griffith J. F. Andersen G. L. (2012). Application of phylogenetic microarray analysis to discriminate sources of fecal pollution. *Environ. Sci. Technol.* 46 4340–4347. 10.1021/es2040366 22360280

[B49] Dunton K. H. Weingartner T. Carmack E. C. (2006). The nearshore western Beaufort Sea ecosystem: circulation and importance of terrestrial carbon in arctic coastal food webs. *Prog. Oceanogr.* 71 362–378. 10.1016/j.pocean.2006.09.011

[B50] Durbin A. M. Teske A. (2011). Microbial diversity and stratification of South Pacific abyssal marine sediments. *Environ. Microbiol.* 13 3219–3234. 10.1111/j.1462-2920.2011.02544.x 21895908

[B51] Dyksma S. Lenk S. Sawicka J. E. Mußmann M. (2018). Uncultured gammaproteobacteria and desulfobacteraceae account for major acetate assimilation in a Coastal Marine Sediment. *Front. Microbiol.* 9:3124. 10.3389/fmicb.2018.03124 30619197 PMC6305295

[B52] Edgar R. C. (2018). Updating the 97% identity threshold for 16S ribosomal RNA OTUs. *Bioinformatics* 34 2371–2375. 10.1093/bioinformatics/bty113 29506021

[B53] Eert J. Meisterhans G. Michel C. Niemi A. Reist J. Williams W. J. (2015). *Physical, Chemical and Biological Oceanographic Data from the Beaufort Regional Environmental Assessment: Marine Fishes Project, August- September 2012.* Ottawa: Fisheries and Oceans Canada.

[B54] Evans P. N. Parks D. H. Chadwick G. L. Robbins S. J. Orphan V. J. Golding S. D. (2015). Methane metabolism in the archaeal phylum Bathyarchaeota revealed by genome-centric metagenomics. *Science* 350 434–438. 10.1126/science.aac7745 26494757

[B55] Forest A. Bélanger S. Sampei M. Sasaki H. Lalande C. Fortier L. (2010). Three-year assessment of particulate organic carbon fluxes in Amundsen Gulf (Beaufort Sea): satellite observations and sediment trap measurements. *Deep. Res. Part I Oceanogr. Res. Pap.* 57 125–142. 10.1016/j.dsr.2009.10.002

[B56] Fry J. C. Webster G. Cragg B. A. Weightman A. J. Parkes R. J. (2006). Analysis of DGGE profiles to explore the relationship between prokaryotic community composition and biogeochemical processes in deep subseafloor sediments from the Peru Margin. *FEMS Microbiol. Ecol.* 58 86–98. 10.1111/j.1574-6941.2006.00144.x 16958910

[B57] Fuhrman J. A. (2009). Microbial community structure and its functional implications. *Nature* 459 193–199. 10.1038/nature08058 19444205

[B58] Fuhrman J. A. Cram J. A. Needham D. M. (2015). Marine microbial community dynamics and their ecological interpretation. *Nat. Rev. Microbiol.* 13 133–146. 10.1038/nrmicro3417 25659323

[B59] Fujii T. Sugino H. Rouse J. D. Furukawa K. (2002). Characterization of the microbial community in an anaerobic ammonium-oxidizing biofilm cultured on a nonwoven biomass carrier. *J. Biosci. Bioeng.* 94 412–418. 10.1016/S1389-1723(02)80218-3 16233327

[B60] Galand P. E. Potvin M. Casamayor E. O. Lovejoy C. (2010). Hydrography shapes bacterial biogeography of the deep Arctic Ocean. *ISME J.* 4 564–576. 10.1038/ismej.2009.134 20010630

[B61] Gamboa A. Montero-Serrano J.-C. St-Onge G. Rochon A. Desiage P.-A. (2017). Mineralogical, geochemical, and magnetic signatures of surface sediments from the Canadian Beaufort Shelf and Amundsen Gulf (*Canadian Arctic*). *Geochem. Geophys. Geosyst.* 18 488–512. 10.1002/2016GC006477

[B62] Garrity G. M. Bell J. A. Lilburn T. (2015). “Thiotrichaceae fam. nov,” in *Bergey’s Manual of Systematics of Archaea and Bacteria*, eds Kim S. B. Goodfellow M. (Chichester, UK: John Wiley & Sons, Ltd).

[B63] Glöckner F. O. Yilmaz P. Quast C. Gerken J. Beccati A. Ciuprina A. (2017). 25 years of serving the community with ribosomal RNA gene reference databases and tools. *J. Biotechnol.* 261 169–176. 10.1016/j.jbiotec.2017.06.1198 28648396

[B64] Goñi M. A. O’Connor A. E. Kuzyk Z. Z. Yunker M. B. Gobeil C. Macdonald R. W. (2013). Distribution and sources of organic matter in surface marine sediments across the North American Arctic margin. *J. Geophys. Res. Ocean.* 118 4017–4035. 10.1002/jgrc.20286

[B65] Gonzalez J. M. Fernandez-Gomez B. Fernandez-Guerra A. Gomez-Consarnau L. Sanchez O. Coll-Llado M. (2008). Genome analysis of the proteorhodopsin-containing marine bacterium *Polaribacter* sp. MED152 (Flavobacteria). *Proc. Natl. Acad. Sci. U.S.A.* 105 8724–8729. 10.1073/pnas.0712027105 18552178 PMC2438413

[B66] Gosink J. J. Herwig R. P. Staley J. T. (1997). *Octadecabacter arcticus* gen. nov., sp. nov., and *O. antarcticus*, sp. nov., nonpigmented, psychrophilic gas vacuolate bacteria from polar sea ice and water. *Syst. Appl. Microbiol.* 20 356–365. 10.1016/S0723-2020(97)80003-3

[B67] Gosink J. J. Woese C. R. Staley J. T. (1998). Polaribacter gen. nov., with three new species, *P. irgensii* sp. nov., *P. franzmannii* sp. nov. and *P. filamentus* sp. nov., gas vacuolate polar marine bacteria of the Cytophaga-Flavobacterium-Bacteroides group and reclassification of *Flectobacillus glomera*. *Int. J. Syst. Bacteriol.* 48 223–235. 10.1099/00207713-48-1-223 9542092

[B68] Grahn N. Olofsson M. Ellnebo-Svedlund K. Monstein H.-J. Jonasson J. (2003). Identification of mixed bacterial DNA contamination in broad-range PCR amplification of 16S rDNA V1 and V3 variable regions by pyrosequencing of cloned amplicons. *FEMS Microbiol. Lett.* 219 87–91. 10.1016/S0378-1097(02)01190-4 12594028

[B69] Grebmeier J. M. (2012). Shifting patterns of life in the pacific arctic and sub-arctic seas. *Ann. Rev. Mar. Sci.* 4 63–78. 10.1146/annurev-marine-120710-100926 22457969

[B70] Grossart H.-P. Levold F. Allgaier M. Simon M. Brinkhoff T. (2005). Marine diatom species harbour distinct bacterial communities. *Environ. Microbiol.* 7 860–873. 10.1111/j.1462-2920.2005.00759.x 15892705

[B71] Hamdan L. J. Coffin R. B. Sikaroodi M. Greinert J. Treude T. Gillevet P. M. (2013). Ocean currents shape the microbiome of Arctic marine sediments. *ISME J.* 7 685–696. 10.1038/ismej.2012.143 23190727 PMC3603395

[B72] Han Y. Perner M. (2015). The globally widespread genus Sulfurimonas: versatile energy metabolisms and adaptations to redox clines. *Front. Microbiol.* 6:989. 10.3389/fmicb.2015.00989 26441918 PMC4584964

[B73] Head I. M. Jones D. M. Röling W. F. M. (2006). Marine microorganisms make a meal of oil. *Nat. Rev. Microbiol.* 4 173–182. 10.1038/nrmicro1348 16489346

[B74] Helbert W. (2017). Marine polysaccharide sulfatases. *Front. Mar. Sci.* 4:6 10.3389/fmars.2017.00006

[B75] Hoffmann K. Hassenrück C. Salman-Carvalho V. Holtappels M. Bienhold C. (2017). Response of bacterial communities to different detritus compositions in arctic deep-sea sediments. *Front. Microbiol.* 8:266. 10.3389/fmicb.2017.00266 28286496 PMC5323390

[B76] Holmes R. M. Coe M. T. Fiske G. J. Gurtovaya T. McClelland J. W. Shiklomanov A. I. (2012). “Climate change impacts on the hydrology and biogeochemistry of arctic rivers,” in *Climatic Change and Global Warming of Inland Waters: Impacts and Mitigation for Ecosystems and Societies*, eds Kumagai M. Goldman C. R. Robarts R. D. (Chichester, UK: John Wiley & Sons, Ltd), 1–26.

[B77] Holmes R. M. McClelland J. W. Peterson B. J. Shiklomanov I. A. Shiklomanov A. I. Zhulidov A. V. (2002). A circumpolar perspective on fluvial sediment flux to the Arctic ocean. *Glob. Biogeochem. Cycles* 16 45–41. 10.1029/2001GB001849

[B78] Hubert C. Arnosti C. Brüchert V. Loy A. Vandieken V. Jørgensen B. B. (2010). Thermophilic anaerobes in Arctic marine sediments induced to mineralize complex organic matter at high temperature. *Environ. Microbiol.* 12 1089–1104. 10.1111/j.1462-2920.2010.02161.x 20192966

[B79] Inagaki F. Suzuki M. Takai K. Oida H. Sakamoto T. Aoki K. (2003). Microbial communities associated with geological horizons in coastal subseafloor sediments from the Sea of Okhotsk. *Appl. Environ. Microbiol.* 69 7224–7235. 10.1128/AEM.69.12.7224-7235.2003 14660370 PMC309994

[B80] Jacob M. Soltwedel T. Boetius A. Ramette A. (2013). Biogeography of deep-sea benthic bacteria at regional scale (Lter Hausgarten, Fram Strait, Arctic). *PLoS One* 8:e72779. 10.1371/journal.pone.0072779 24023770 PMC3759371

[B81] Jørgensen B. Glud R. Holby O. (2005). Oxygen distribution and bioirrigation in Arctic fjord sediments (Svalbard, Barents Sea). *Mar. Ecol. Prog. Ser.* 292 85–95. 10.3354/meps292085

[B82] Jørgensen B. B. Findlay A. J. Pellerin A. (2019). The biogeochemical sulfur cycle of marine sediments. *Front. Microbiol.* 10:849. 10.3389/fmicb.2019.00849 31105660 PMC6492693

[B83] Jorgensen S. L. Hannisdal B. Lanzén A. Baumberger T. Flesland K. (2012). Correlating microbial community profiles with geochemical data in highly stratified sediments from the Arctic Mid-Ocean Ridge. *Proc. Natl. Acad. Sci. U.S.A.* 109 16764–16765. 10.1594/PANGAEA.786687 PMC3479504 23027979

[B84] Junge K. Imhoff F. Staley T. Deming W. (2002). Phylogenetic diversity of numerically important arctic sea-ice bacteria cultured at subzero temperature. *Microb. Ecol.* 43 315–328. 10.1007/s00248-001-1026-4 12037610

[B85] Kêdra M. Moritz C. Choy E. S. David C. Degen R. Duerksen S. (2015). Status and trends in the structure of Arctic benthic food webs. *Polar Res.* 34:23775 10.3402/polar.v34.23775

[B86] Kirchman D. L. Hanson T. E. Cottrell M. T. Hamdan L. J. (2014). Metagenomic analysis of organic matter degradation in methane-rich Arctic Ocean sediments. *Limnol. Oceanogr.* 59 548–559. 10.4319/lo.2014.59.2.0548

[B87] Kleindienst S. Herbst F. A. Stagars M. Von Netzer F. Von Bergen M. Seifert J. (2014). Diverse sulfate-reducing bacteria of the Desulfosarcina/*Desulfococcus clade* are the key alkane degraders at marine seeps. *ISME J.* 8 2029–2044. 10.1038/ismej.2014.51 24722631 PMC4184016

[B88] Kleindienst S. Ramette A. Amann R. Knittel K. (2012). Distribution and in situ abundance of sulfate-reducing bacteria in diverse marine hydrocarbon seep sediments. *Environ. Microbiol.* 14 2689–2710. 10.1111/j.1462-2920.2012.02832.x 22882476

[B89] Koedooder C. Stock W. Willems A. Mangelinckx S. De Troch M. Vyverman W. (2019). Diatom-bacteria interactions modulate the composition and productivity of benthic diatom biofilms. *Front. Microbiol.* 10:1255. 10.3389/fmicb.2019.01255 31231340 PMC6561236

[B90] Könneke M. Schubert D. M. Brown P. C. Hügler M. Standfest S. Schwander T. (2014). Ammonia-oxidizing archaea use the most energy-efficient aerobic pathway for CO2 fixation. *Proc. Natl. Acad. Sci. U.S.A.* 111 8239–8244. 10.1073/pnas.1402028111 24843170 PMC4050595

[B91] Kortsch S. Primicerio R. Fossheim M. Dolgov A. V. Aschan M. (2015). Climate change alters the structure of arctic marine food webs due to poleward shifts of boreal generalists. *Proc. R. Soc. B Biol. Sci.* 282:20151546. 10.1098/rspb.2015.1546 26336179 PMC4571709

[B92] Kostka J. Thamdrup B. Glud R. Canfield D. (1999). Rates and pathways of carbon oxidation in permanently cold Arctic sediments. *Mar. Ecol. Prog. Ser.* 180 7–21. 10.3354/meps180007

[B93] Kostka J. E. Prakash O. Overholt W. A. Green S. J. Freyer G. Canion A. (2011). Hydrocarbon-degrading bacteria and the bacterial community response in Gulf of Mexico beach sands impacted by the deepwater horizon oil spill. *Appl. Environ. Microbiol.* 77 7962–7974. 10.1128/AEM.05402-11 21948834 PMC3208977

[B94] Kuever J. (2014a). “The Family Desulfobacteraceae,” in *The Prokaryotes*, eds Balows A. Trüper H.G. Dworkin M. Harder W. Schleifer K.-H. (Berlin: Springer), 45–73. 10.1007/978-3-642-39044-9_266

[B95] Kuever J. (2014b). “The Family Desulfobulbaceae,” in *The Prokaryotes*, eds Balows A. Trüper H.G. Dworkin M. Harder W. Schleifer K.-H. (Berlin: Springer), 75–86. 10.1007/978-3-642-39044-9_267

[B96] Kulakov L. A. McAlister M. B. Ogden K. L. Larkin M. J. O’Hanlon J. F. (2002). Analysis of bacteria contaminating ultrapure water in industrial systems. *Appl. Environ. Microbiol.* 68 1548–1555. 10.1128/aem.68.4.1548-1555.2002 11916667 PMC123900

[B97] Landry Z. Swa B. K. Herndl G. J. Stepanauskas R. Giovannoni S. J. (2017). SAR202 genomes from the dark ocean predict pathways for the oxidation of recalcitrant dissolved organic matter. *mBio* 8:e00413-17. 10.1128/mBio.00413-17 28420738 PMC5395668

[B98] Lansard B. Mucci A. Miller L. A. Macdonald R. W. Gratton Y. (2012). Seasonal variability of water mass distribution in the southeastern Beaufort Sea determined by total alkalinity and δ 18 O. *J. Geophys. Res. Ocean* 117:JC007299 10.1029/2011JC007299

[B99] Laurence M. Hatzis C. Brash D. E. (2014). Common contaminants in next-generation sequencing that hinder discovery of low-abundance microbes. *PLoS One* 9:e97876. 10.1371/journal.pone.0097876 24837716 PMC4023998

[B100] Lee D. H. Kim J. H. Lee Y. M. Stadnitskaia A. Jin Y. K. Niemann H. (2018). Biogeochemical evidence of anaerobic methane oxidation on active submarine mud volcanoes on the continental slope of the Canadian Beaufort Sea. *Biogeosciences* 15 7419–7433. 10.5194/bg-15-7419-2018

[B101] Lee Y. M. Jung Y.-J. Hong S. G. Kim J. H. Lee H. K. (2014). Diversity and Physiological Characteristics of Culturable Bacteria from Marine Sediments of Ross Sea, Antarctica. *Korean J. Microbiol.* 50 119–127. 10.7845/kjm.2014.4014

[B102] Lefevre C. T. Bazylinski D. A. (2013). Ecology, diversity, and evolution of magnetotactic bacteria. *Microbiol. Mol. Biol. Rev.* 77 497–526. 10.1128/mmbr.00021-13 24006473 PMC3811606

[B103] Li H. Yu Y. Luo W. Zeng Y. Chen B. (2009). Bacterial diversity in surface sediments from the Pacific Arctic Ocean. *Extremophiles* 13 233–246. 10.1007/s00792-009-0225-7 19153801

[B104] Li H. Zhang X.-Y. Liu C. Lin C.-Y. Xu Z. Chen X.-L. (2014). *Polaribacter huanghezhanensis* sp. nov., isolated from Arctic fjord sediment, and emended description of the genus Polaribacter. *Int. J. Syst. Evol. Microbiol.* 64 973–978. 10.1099/ijs.0.056788-0 24425815

[B105] Li Y. Liu Q. Li C. Dong Y. Zhang W. Zhang W. (2015). Bacterial and archaeal community structures in the Arctic deep-sea sediment. *Acta Oceanol. Sin.* 34 93–113. 10.1007/s13131-015-0624-9

[B106] Liang B. Wang L.-Y. Mbadinga S. M. Liu J.-F. Yang S.-Z. Gu J.-D. (2015). Anaerolineaceae and Methanosaeta turned to be the dominant microorganisms in alkanes-dependent methanogenic culture after long-term of incubation. *AMB Express* 5:37. 10.1186/s13568-015-0117-4 26080793 PMC4469597

[B107] Lopez-Fernandez M. Simone D. Wu X. Soler L. Nilsson E. Holmfeldt K. (2018). Metatranscriptomes reveal that all three domains of life are active but are dominated by bacteria in the fennoscandian crystalline granitic continental deep biosphere. *mBio* 9:e01792-18. 10.1128/mBio.01792-18 30459191 PMC6247080

[B108] Lorenson T. D. Grienert J. Coffin R. B. (2016). Dissolved methane in the Beaufort Sea and the Arctic Ocean, 1992–2009; sources and atmospheric flux. *Limnol. Oceanogr.* 61 S300–S323. 10.1002/lno.10457

[B109] Losey N. A. Stevenson B. S. Busse H.-J. Damste J. S. S. Rijpstra W. I. C. Rudd S. (2013). *Thermoanaerobaculum aquaticum* gen. nov., sp. nov., the first cultivated member of Acidobacteria subdivision 23, isolated from a hot spring. *Int. J. Syst. Evol. Microbiol.* 63 4149–4157. 10.1099/ijs.0.051425-0 23771620

[B110] Macdonald R. W. Solomon S. M. Cranston R. E. Welch H. E. Yunker M. B. Gobeil C. (1998). A sediment and organic carbon budget for the Canadian Beaufort Shelf. *Mar. Geol.* 144 255–273. 10.1016/S0025-3227(97)00106-0

[B111] Magen C. Chaillou G. Crowe S. A. Mucci A. Sundby B. Gao A. (2010). Origin and fate of particulate organic matter in the southern Beaufort Sea - Amundsen Gulf region, Canadian Arctic. *Estuar. Coast. Shelf Sci.* 86 31–41. 10.1016/j.ecss.2009.09.009

[B112] Martens-Habbena W. Qin W. Horak R. E. A. Urakawa H. Schauer A. J. Moffett J. W. (2015). The production of nitric oxide by marine ammonia-oxidizing archaea and inhibition of archaeal ammonia oxidation by a nitric oxide scavenger. *Environ. Microbiol.* 17 2261–2274. 10.1111/1462-2920.12677 25420929

[B113] McBride M. J. (2014). “The Family Flavobacteriaceae,” in *The Prokaryotes*, eds Balows A. Trüper H.G. Dworkin M. Harder W. Schleifer K.-H. (Berlin: Springer), 643–676.

[B114] McIlroy S. J. Kirkegaard R. H. Dueholm M. S. Fernando E. Karst S. M. Albertsen M. (2017). Culture-independent analyses reveal novel anaerolineaceae as abundant primary fermenters in anaerobic digesters treating waste activated sludge. *Front. Microbiol.* 8:1134. 10.3389/fmicb.2017.01134 28690595 PMC5481317

[B115] McTigue N. D. Gardner W. S. Dunton K. H. Hardison A. K. (2016). Biotic and abiotic controls on co-occurring nitrogen cycling processes in shallow Arctic shelf sediments. *Nat. Commun.* 7:13145. 10.1038/ncomms13145 27782213 PMC5095177

[B116] Miquel J. C. Gasser B. Martín J. Marec C. Babin M. Fortier L. (2015). Downward particle flux and carbon export in the Beaufort Sea, Arctic Ocean; the role of zooplankton. *Biogeosciences* 12 5103–5117. 10.5194/bg-12-5103-2015

[B117] Morris E. K. Caruso T. Buscot F. Fischer M. Hancock C. Maier T. S. (2014). Choosing and using diversity indices: insights for ecological applications from the German Biodiversity Exploratories. *Ecol. Evol.* 4 3514–3524. 10.1002/ece3.1155 25478144 PMC4224527

[B118] Morris R. M. Rappé M. S. Urbach E. Connon S. A. Giovannoni S. J. (2004). Prevalence of the Chloroflexi-related SAR202 bacterioplankton cluster throughout the mesopelagic zone and deep ocean. *Appl. Environ. Microbiol.* 70 2836–2842. 10.1128/AEM.70.5.2836-2842.2004 15128540 PMC404461

[B119] Müller A. L. de Rezende J. R. Hubert C. R. J. Kjeldsen K. U. Lagkouvardos I. Berry D. (2014). Endospores of thermophilic bacteria as tracers of microbial dispersal by ocean currents. *ISME J.* 8 1153–1165. 10.1038/ismej.2013.225 24351936 PMC4030223

[B120] Müller A. L. Pelikan C. de Rezende J. R. Wasmund K. Putz M. Glombitza C. (2018). Bacterial interactions during sequential degradation of cyanobacterial necromass in a sulfidic arctic marine sediment. *Environ. Microbiol.* 20 2927–2940. 10.1111/1462-2920.14297 30051650 PMC6175234

[B121] Naidu A. S. Cooper L. W. Finney B. P. Macdonald R. W. Alexander C. Semiletov I. P. (2000). Organic carbon isotope ratio (δ13C) of Arctic Amerasian Continental shelf sediments. *Int. J. Earth Sci.* 89 522–532. 10.1007/s005310000121

[B122] Nelson R. J. Ashjian C. J. Bluhm B. A. Conlan K. E. Gradinger R. R. Grebmeier J. M. (2014). “Biodiversity and biogeography of the lower trophic taxa of the pacific arctic region: sensitivities to climate change,” in *The Pacific Arctic Region: Ecosystem Status and Trends in a Rapidly Changing Environment*, eds Masłowski W. Grebmeier J. M. (Netherlands: Springer), 269–336.

[B123] Niemi A. Michel C. Dempsey M. Eert J. Reist J. Williams W. J. (2015). *Physical, Chemical and Biological Oceanographic Data From the Beaufort Regional Environmental Assessment: Marine Fishes Project, August- September 2013.* Ottawa: Fisheries and Oceans Canada.

[B124] Nunoura T. Nishizawa M. Hirai M. Shimamura S. Harnvoravongchai P. Koide O. (2018). Microbial diversity in sediments from the bottom of the challenger deep, the mariana trench. *Microbes Environ.* 33 186–194. 10.1264/jsme2.ME17194 29806625 PMC6031389

[B125] Obi C. C. Adebusoye S. A. Amund O. O. Ugoji E. O. Ilori M. O. Hedman C. J. (2017). Structural dynamics of microbial communities in polycyclic aromatic hydrocarbon-contaminated tropical estuarine sediments undergoing simulated aerobic biotreatment. *Appl. Microbiol. Biotechnol.* 101 4299–4314. 10.1007/s00253-017-8151-6 28190100

[B126] Offre P. Spang A. Schleper C. (2013). Archaea in biogeochemical cycles. *Annu. Rev. Microbiol.* 67 437–457. 10.1146/annurev-micro-092412-155614 23808334

[B127] Oksanen J. (2015). *Multivariate Analysis of Ecological Communities in R: Vegan Tutorial.* Available online at: http://cc.oulu.fi/~jarioksa/opetus/metodi/vegantutor.pdf (accessed December 13, 2017).

[B128] Oksanen J. Blanchet F. G. Friendly M. Kindt R. Legendre P. Mcglinn D. (2017). *Title Community Ecology Package.* Available online at: https://github.com/vegandevs/vegan/issues (accessed December 13, 2017).

[B129] Orcutt B. N. Sylvan J. B. Knab N. J. Edwards K. J. (2011). Microbial ecology of the dark ocean above, at, and below the seafloor. *Microbiol. Mol. Biol. Rev.* 75 361–422. 10.1128/MMBR.00039-10 21646433 PMC3122624

[B130] Orphan V. J. Hinrichs K. U. Ussler W. Paull C. K. Taylor L. T. Sylva S. P. (2001). Comparative analysis of methane-oxidizing archaea and sulfate-reducing bacteria in anoxic marine sediments. *Appl. Environ. Microbiol.* 67 1922–1934. 10.1128/AEM.67.4.1922-1934.2001 11282650 PMC92814

[B131] Otte J. M. Blackwell N. Ruser R. Kappler A. Kleindienst S. Schmidt C. (2019). N2O formation by nitrite-induced (chemo)denitrification in coastal marine sediment. *Sci. Rep.* 9:10691. 10.1038/s41598-019-47172-x 31366952 PMC6668465

[B132] Parada A. E. Needham D. M. Fuhrman J. A. (2016). Every base matters: assessing small subunit rRNA primers for marine microbiomes with mock communities, time series and global field samples. *Environ. Microbiol.* 18 1403–1414. 10.1111/1462-2920.13023 26271760

[B133] Park B. J. Park S. J. Yoon D. N. Schouten S. Damsté J. S. S. Rhee S. K. (2010). Cultivation of autotrophic ammonia-oxidizing archaea from marine sediments in coculture with sulfur-oxidizing bacteria. *Appl. Environ. Microbiol.* 76 7575–7587. 10.1128/AEM.01478-10 20870784 PMC2976178

[B134] Park S. J. Kim J. G. Jung M. Y. Kim S. J. Cha I. T. Ghai R. (2012). Draft genome sequence of an ammonia-oxidizing archaeon, “Candidatus Nitrosopumilus sediminis” AR2, from svalbard in the arctic circle. *J. Bacteriol.* 194 6946–6947. 10.1128/JB.01869-12 23209211 PMC3510607

[B135] Paull C. Dallimore S. Hughes-Clarke J. Blasco S. Lundsten E. Iii W. U. (2011). *Tracking the Decomposition of Submarine Permafrost and Gas Hydrate Under the Shelf and Slope of the Beaufort Sea.* Moss Landing, CA: Monterey Bay Aquarium Research Institute (MBARI).

[B136] Paull C. K. Dallimore S. R. Caress D. W. Gwiazda R. Melling H. Riedel M. (2015). Active mud volcanoes on the continental slope of the Canadian Beaufort Sea. *Geochem. Geophys. Geosyst.* 16 3160–3181. 10.1002/2015GC005928

[B137] Penton C. R. Devol A. H. Tiedje J. M. (2006). Molecular evidence for the broad distribution of anaerobic ammonium-oxidizing bacteria in freshwater and marine sediments. *Appl. Environ. Microbiol.* 72 6829–6832. 10.1128/AEM.01254-06 17021238 PMC1610322

[B138] Petro C. Jochum L. M. Schreiber L. Marshall I. P. G. Schramm A. Kjeldsen K. U. (2019). Single-cell amplified genomes of two uncultivated members of the deltaproteobacterial SEEP-SRB1 clade, isolated from marine sediment. *Mar. Genomics* 46 66–69. 10.1016/j.margen.2019.01.004

[B139] Pickart R. S. (2004). Shelfbreak circulation in the Alaskan Beaufort Sea: mean structure and variability. *J. Geophys. Res. C Ocean.* 109 1–14. 10.1029/2003JC001912

[B140] Pjevac P. Kamyshny A. Dyksma S. Mußmann M. (2014). Microbial consumption of zero-valence sulfur in marine benthic habitats. *Environ. Microbiol.* 16 3416–3430. 10.1111/1462-2920.12410 24467476

[B141] Probandt D. Knittel K. Tegetmeyer H. E. Ahmerkamp S. Holtappels M. Amann R. (2017). Permeability shapes bacterial communities in sublittoral surface sediments. *Environ. Microbiol.* 19 1584–1599. 10.1111/1462-2920.13676 28120371

[B142] Qin W. Amin S. A. Martens-Habbena W. Walker C. B. Urakawa H. Devol A. H. (2014). Marine ammonia-oxidizing archaeal isolates display obligate mixotrophy and wide ecotypic variation. *Proc. Natl. Acad. Sci. U.S.A.* 111 12504–12509. 10.1073/pnas.1324115111 25114236 PMC4151751

[B143] Qin W. Martens-Habbena W. Kobelt J. N. Stahl D. A. (2016). “Candidatus nitrosopumilales,” in *Bergey’s Manual of Systematics of Archaea and Bacteria*, eds Kim S. B. Goodfellow M. (Chichester, UK: John Wiley & Sons, Ltd), 1–2.

[B144] R Core Team (2017). *R: A Language and Environment for Statistical Computing.* Vienna: R Foundation for Statistical Computing.

[B145] Rachold V. Eicken H. Gordeev V. V. Grigoriev M. N. Hubberten H. (2004). The organic carbon cycle in the Arctic Ocean. *Org. Carbon Cycle Arct. Ocean* 363 33–55. 10.1007/978-3-642-18912-8

[B146] Rapp J. Z. Fernández-Méndez M. Bienhold C. Boetius A. (2018). Effects of Ice-Algal Aggregate Export on the Connectivity of Bacterial Communities in the Central Arctic Ocean. *Front. Microbiol.* 9:1035. 10.3389/fmicb.2018.01035 29875749 PMC5974969

[B147] Ravenschlag K. Sahm K. Amann R. (2001). Quantitative molecular analysis of the microbial community in marine arctic sediments (svalbard) quantitative molecular analysis of the microbial community in marine arctic sediments (Svalbard). *Appl. Environ. Microbiol.* 67 387–395. 10.1128/AEM.67.1.387 11133470 PMC92590

[B148] Ravenschlag K. Sahm K. Knoblauch C. Jørgensen B. B. Amann R. (2000). Community structure, cellular rRNA content, and activity of sulfate-reducing bacteria in marine arctic sediments. *Appl. Environ. Microbiol.* 66 3592–3602. 10.1128/aem.66.8.3592-3602.2000 10919825 PMC92189

[B149] Renaud P. E. Wallhead P. Kotta J. Włodarska-Kowalczuk M. Bellerby R. G. J. Rätsep M. (2019). Arctic Sensitivity? Suitable habitat for benthic taxa is surprisingly robust to climate change. *Front. Mar. Sci.* 6:538 10.3389/fmars.2019.00538

[B150] Retelletti Brogi S. Kim J.-H. Ryu J.-S. Jin Y. K. Lee Y. K. Hur J. (2019). Exploring sediment porewater dissolved organic matter (DOM) in a mud volcano: clues of a thermogenic DOM source from fluorescence spectroscopy. *Mar. Chem.* 211:9 10.1016/J.MARCHEM.2019.03.009

[B151] Roussel E. G. Sauvadet A.-L. Chaduteau C. Fouquet Y. Charlou J.-L. Prieur D. (2009). Archaeal communities associated with shallow to deep subseafloor sediments of the New Caledonia Basin. *Environ. Microbiol.* 11 2446–2462. 10.1111/j.1462-2920.2009.01976.x 19624712

[B152] Ruff S. E. Biddle J. F. Teske A. P. Knittel K. Boetius A. Ramette A. (2015). Global dispersion and local diversification of the methane seep microbiome. *Proc. Natl. Acad. Sci. U.S.A.* 112 4015–4020. 10.1073/pnas.1421865112 25775520 PMC4386351

[B153] Rysgaard S. Glud R. N. Risgaard-Petersen N. Dalsgaard T. (2004). Denitrification and anammox activity in Arctic marine sediments. *Limnol. Oceanogr.* 49 1493–1502. 10.4319/lo.2004.49.5.1493

[B154] Salter S. J. Cox M. J. Turek E. M. Calus S. T. Cookson W. O. Moffatt M. F. (2014). Reagent and laboratory contamination can critically impact sequence-based microbiome analyses. *BMC Biol.* 12:87. 10.1186/s12915-014-0087-z 25387460 PMC4228153

[B155] Schloss P. D. Westcott S. L. Ryabin T. Hall J. R. Hartmann M. Hollister E. B. (2009). Introducing mothur: open-source, platform-independent, community-supported software for describing and comparing microbial communities. *Appl. Environ. Microbiol.* 75 7537–7541. 10.1128/AEM.01541-09 19801464 PMC2786419

[B156] Schreiber L. Holler T. Knittel K. Meyerdierks A. Amann R. (2010). Identification of the dominant sulfate-reducing bacterial partner of anaerobic methanotrophs of the ANME-2 clade. *Environ. Microbiol.* 12 2327–2340. 10.1111/j.1462-2920.2010.02275.x 21966923

[B157] Sheik C. S. Jain S. Dick G. J. (2014). Metabolic flexibility of enigmatic SAR324 revealed through metagenomics and metatranscriptomics. *Environ. Microbiol.* 16 304–317. 10.1111/1462-2920.12165 23809230

[B158] Siegert M. Krüger M. Teichert B. Wiedicke M. Schippers A. (2011). Anaerobic oxidation of methane at a marine methane seep in a forearc sediment basin off sumatra, Indian Ocean. *Front. Microbiol.* 2:249. 10.3389/fmicb.2011.00249 22207865 PMC3245565

[B159] Simpson K. G. Tremblay J. -É Gratton Y. Price N. M. (2008). An annual study of inorganic and organic nitrogen and phosphorus and silicic acid in the southeastern Beaufort Sea. *J. Geophys. Res.* 113:C07016 10.1029/2007JC004462

[B160] Sinkko H. Lukkari K. Sihvonen L. M. Sivonen K. Leivuori M. Rantanen M. (2013). Bacteria contribute to sediment nutrient release and reflect progressed eutrophication-driven hypoxia in an organic-rich continental Sea. *PLoS One* 8:e67061. 10.1371/journal.pone.0067061 23825619 PMC3692436

[B161] Smoot C. A. Hopcroft R. R. (2017). Depth-stratified community structure of Beaufort Sea slope zooplankton and its relations to water masses. *J. Plankton Res.* 39 79–91. 10.1093/plankt/fbw087 32665766

[B162] Sørensen K. B. Teske A. (2006). Stratified communities of active archaea in deep marine subsurface sediments. *Appl. Environ. Microbiol.* 72 4596–4603. 10.1128/AEM.00562-06 16820449 PMC1489303

[B163] Sparrow K. J. Kessler J. D. Southon J. R. Garcia-Tigreros F. Schreiner K. M. Ruppel C. D. (2018). Limited contribution of ancient methane to surface waters of the U.S. Beaufort Sea shelf. *Sci. Adv.* 4:eaao4842. 10.1126/sciadv.aao4842 29349299 PMC5771695

[B164] Stahl D. A. de la Torre J. R. (2012). Physiology and diversity of ammonia-oxidizing archaea. *Annu. Rev. Microbiol.* 66 83–101. 10.1146/annurev-micro-092611-150128 22994489

[B165] Stokes C. R. Clark C. D. Winsborrow M. C. M. (2006). Subglacial bedform evidence for a major palaeo-ice stream and its retreat phases in Amundsen Gulf, Canadian arctic archipelago. *J. Quat. Sci.* 21 399–412. 10.1002/jqs.991

[B166] Takeuchi M. Katayama T. Yamagishi T. Hanada S. Tamaki H. Kamagata Y. (2014). *Methyloceanibacter caenitepidi* gen. nov., sp. nov., a facultatively methylotrophic bacterium isolated from marine sediments near a hydrothermal vent. *Int. J. Syst. Evol. Microbiol.* 64 462–468. 10.1099/ijs.0.053397-0 24096357

[B167] Tanaka N. Romanenko L. A. Iino T. Frolova G. M. Mikhailov V. V. (2011). *Cocleimonas flava* gen. nov., sp. nov., a gammaproteobacterium isolated from sand snail (*Umbonium costatum*). *Int. J. Syst. Evol. Microbiol.* 61 412–416. 10.1099/ijs.0.020263-0 20348322

[B168] Teeling H. Fuchs B. M. Becher D. Klockow C. Gardebrecht A. Bennke C. M. (2012). Substrate-controlled succession of marine bacterioplankton populations induced by a phytoplankton bloom. *Science* 336 608–611. 10.1126/science.1218344 22556258

[B169] Teeling H. Fuchs B. M. Bennke C. M. Krüger K. Chafee M. Kappelmann L. (2016). Recurring patterns in bacterioplankton dynamics during coastal spring algae blooms. *eLife* 5:e11888. 10.7554/eLife.11888 27054497 PMC4829426

[B170] Thamdrup B. Dalsgaard T. (2002). Production of N2 through anaerobic ammonium oxidation coupled to nitrate reduction in marine sediments. *Appl. Environ. Microbiol.* 68 1312–1318. 10.1128/AEM.68.3.1312-1318.2002 11872482 PMC123779

[B171] Trembath-Reichert E. Case D. H. Orphan V. J. (2016). Characterization of microbial associations with methanotrophic archaea and sulfate-reducing bacteria through statistical comparison of nested Magneto-FISH enrichments. *PeerJ* 2016:e1913. 10.7717/peerj.1913 27114874 PMC4841229

[B172] Treude T. Krause S. Maltby J. Dale A. W. Coffin R. Hamdan L. J. (2014). Sulfate reduction and methane oxidation activity below the sulfate-methane transition zone in Alaskan Beaufort Sea continental margin sediments: implications for deep sulfur cycling. *Geochim. Cosmochim. Acta* 144 217–237. 10.1016/J.GCA.2014.08.018

[B173] Trivedi C. B. Lau G. E. Grasby S. E. Templeton A. S. Spear J. R. (2018). Low-temperature sulfidic-ice microbial communities, borup fiord pass, canadian high arctic. *Front. Microbiol.* 9:1622. 10.3389/fmicb.2018.01622 30087659 PMC6066561

[B174] Ullman W. J. Aller R. C. (1982). Diffusion coefficients in nearshore marine sediments. *Limnol. Oceanogr.* 27 552–556. 10.4319/lo.1982.27.3.0552

[B175] Van de Vossenberg J. Woebken D. Maalcke W. J. Wessels H. J. C. T. Dutilh B. E. Kartal B. (2013). The metagenome of the marine anammox bacterium “Candidatus Scalindua profunda” illustrates the versatility of this globally important nitrogen cycle bacterium. *Environ. Microbiol.* 15 1275–1289. 10.1111/j.1462-2920.2012.02774.x 22568606 PMC3655542

[B176] van der Waals M. J. Atashgahi S. da Rocha U. N. van der Zaan B. M. Smidt H. Gerritse J. (2017). Benzene degradation in a denitrifying biofilm reactor: activity and microbial community composition. *Appl. Microbiol. Biotechnol.* 101 5175–5188. 10.1007/s00253-017-8214-8 28321487 PMC5486827

[B177] Vekeman B. Kerckhof F.-M. Cremers G. de Vos P. Vandamme P. Boon N. (2016a). New *Methyloceanibacter* diversity from North Sea sediments includes methanotroph containing solely the soluble methane monooxygenase. *Environ. Microbiol.* 18 4523–4536. 10.1111/1462-2920.13485 27501305

[B178] Vekeman B. Speth D. Wille J. Cremers G. De Vos P. Op den Camp H. J. M. (2016b). Genome characteristics of two novel type I methanotrophs enriched from north sea sediments containing exclusively a lanthanide-dependent XoxF5-type methanol dehydrogenase. *Microb. Ecol.* 72 503–509. 10.1007/s00248-016-0808-7 27457652

[B179] Vollmers J. Voget S. Dietrich S. Gollnow K. Smits M. Meyer K. (2013). Poles apart: arctic and antarctic octadecabacter strains share high genome plasticity and a new type of xanthorhodopsin. *PLoS One* 8:e63422. 10.1371/journal.pone.0063422 23671678 PMC3646047

[B180] Waidner L. A. Kirchman D. L. (2007). Aerobic anoxygenic phototrophic bacteria attached to particles in turbid waters of the Delaware and Chesapeake estuaries. *Appl. Environ. Microbiol.* 73 3936–3944. 10.1128/AEM.00592-07 17468276 PMC1932736

[B181] Walsh J. E. Overland J. E. Groisman P. Y. Rudolf B. (2011). Ongoing climate change in the arctic. *Ambio* 40 6–16. 10.1007/s13280-011-0211-z

[B182] Walters W. Hyde E. R. Berg-Lyons D. Ackermann G. Humphrey G. Parada A. (2016). Improved bacterial 16S rRNA gene (V4 and V4-5) and fungal internal transcribed spacer marker gene primers for microbial community surveys. *mSystems* 1:e00009-15. 10.1128/mSystems.00009-15 27822518 PMC5069754

[B183] Wang L. Yu M. Liu Y. Liu J. Wu Y. Li L. (2018). Comparative analyses of the bacterial community of hydrothermal deposits and seafloor sediments across Okinawa Trough. *J. Mar. Syst.* 180 162–172. 10.1016/j.jmarsys.2016.11.012

[B184] Wang Q. Garrity G. M. Tiedje J. M. Cole J. R. (2007). Naive bayesian classifier for rapid assignment of rRNA sequences into the new bacterial taxonomy. *Appl. Environ. Microbiol.* 73 5261–5267. 10.1128/AEM.00062-07 17586664 PMC1950982

[B185] Wang X. Yan Y. Gao D. (2018). The threshold of influent ammonium concentration for nitrate over-accumulation in a one-stage deammonification system with granular sludge without aeration. *Sci. Total Environ.* 634 843–852. 10.1016/j.scitotenv.2018.04.053 29653428

[B186] Wasmund K. Mußmann M. Loy A. (2017). The life sulfuric: microbial ecology of sulfur cycling in marine sediments. *Environ. Microbiol. Rep.* 9 323–344. 10.1111/1758-2229.12538 28419734 PMC5573963

[B187] Weingartner T. Okkonen S. Danielson S. (2005). Circulation and water property variations in the nearshore Alaskan Beaufort Sea. *US Miner. Manag. Serv. Outer Cont. Shelf Study* 28 1–103.

[B188] Westcott S. L. Schloss P. D. (2017). OptiClust, an improved method for assigning amplicon-based sequence data to operational taxonomic units. *mSphere* 2:e00073-17. 10.1128/mSphereDirect.00073-17 28289728 PMC5343174

[B189] Williams T. J. Lefèvre C. T. Zhao W. Beveridge T. J. Bazylinski D. A. (2012). *Magnetospira thiophila* gen. nov., sp. nov., a marine magnetotactic bacterium that represents a novel lineage within the Rhodospirillaceae (Alphaproteobacteria). *Int. J. Syst. Evol. Microbiol.* 62 2443–2450. 10.1099/ijs.0.037697-0 22140150

[B190] Winkel M. Mitzscherling J. Overduin P. P. Horn F. Winterfeld M. Rijkers R. (2018). Anaerobic methanotrophic communities thrive in deep submarine permafrost. *Sci. Rep.* 8:1291. 10.1038/s41598-018-19505-9 29358665 PMC5778128

[B191] Wu C. H. Sercu B. van de Werfhorst L. C. Wong J. deSantis T. Z. Brodie E. L. (2010). Characterization of coastal urban watershed bacterial communities leads to alternative community-based indicators. *PLoS One* 5:e0011285. 10.1371/journal.pone.0011285 20585654 PMC2890573

[B192] Wu H. Liu M. Zhang W. Xiao T. (2014). Phylogenetic analysis of epibacterial communities on the surfaces of four red macroalgae. *J. Ocean Univ. China* 13 1025–1032. 10.1007/s11802-014-2325-y

[B193] Xia Y. Wang Y. Wang Y. Chin F. Y. L. Zhang T. (2016). Cellular adhesiveness and cellulolytic capacity in Anaerolineae revealed by omics-based genome interpretation. *Biotechnol. Biofuels* 9:111. 10.1186/s13068-016-0524-z 27222666 PMC4877987

[B194] Xing P. Hahnke R. L. Unfried F. Markert S. Huang S. Barbeyron T. (2015). Niches of two polysaccharide-degrading Polaribacter isolates from the North Sea during a spring diatom bloom. *ISME J.* 9 1410–1422. 10.1038/ismej.2014.225 25478683 PMC4438327

[B195] Yamada T. Sekiguchi Y. (2018). “Anaerolineaceae,” in *Bergey’s Manual of Systematics of Archaea and Bacteria*, eds Kim S. B. Goodfellow M. (Chichester, UK: John Wiley & Sons, Ltd), 1–5.

[B196] Yang S. Guo B. Shao Y. Mohammed A. Vincent S. Ashbolt N. J. (2019). The value of floc and biofilm bacteria for anammox stability when treating ammonia-rich digester sludge thickening lagoon supernatant. *Chemosphere* 233 472–481. 10.1016/j.chemosphere.2019.05.287 31181494

[B197] Yekutieli D. Benjamini Y. (2002). The control of the false discovery rate in multiple testing under dependency. *Ann. Stat.* 29 1165–1188. 10.1214/aos/1013699998

[B198] Zeng Y.-X. Yu Y. Li H.-R. Luo W. (2017). Prokaryotic community composition in arctic kongsfjorden and sub-arctic northern bering sea sediments as revealed by 454 pyrosequencing. *Front. Microbiol.* 8:2498. 10.3389/fmicb.2017.02498 29312204 PMC5732994

[B199] Zinger L. Amaral-Zettler L. A. Fuhrman J. A. Horner-Devine M. C. Huse S. M. Welch D. B. M. (2011). Global patterns of bacterial beta-diversity in seafloor and seawater ecosystems. *PLoS One* 6:e0024570. 10.1371/journal.pone.0024570 21931760 PMC3169623

[B200] Zinke L. A. Reese B. K. McManus J. Wheat C. G. Orcutt B. N. Amend J. P. (2018). Sediment microbial communities influenced by cool hydrothermal fluid migration. *Front. Microbiol.* 9:1249. 10.3389/fmicb.2018.01249 29951048 PMC6008377

