# Supplementary material for: Patterns in Benthic Microbial Community Structure Across Environmental Gradients in the Beaufort Sea Shelf and Slope
Source: Front Microbiol. 2021 Jan 28;12:581124. doi: 10.3389/fmicb.2021.581124 (PMC7876419; doi:10.3389/fmicb.2021.581124)
Supplement: Supplementary Table 1 — Indicator taxa for each prokaryotic assemblage. Taxa are arranged from most abundant starting at the top to least abundant toward the bottom. Those without a semi-colon indicate OTUs which could not be IDed past the taxonomic level provided. [file Table_1.DOCX]

| **A** | **B** | **C** | **D** |
| --- | --- | --- | --- |
| NB1-J | - | Rhodobacteraceae | Anaerolineaceae |
| Nitrosopumilaceae; *Nitrosopumilus* | - | Flavobacteriaceae | Desulfobacteraceae |
| Subgroup 22 | - | Flavobacteriaceae; *Ulvibacter* | 4572-13 |
| Nitrosopumilaceae | - | Saprospiraceae | Anaerolineaceae |
| Thermoanaerobaculaceae; Subgroup 10 | - | Alphaproteobacteria | Bacteroidetes BD2-2 |
| Alphaproteobacteria | - | Flavobacteriaceae; *Lutibacter* | Bathyarchaeota |
| Gammaproteobacteria  Incertae Sedis | - | Flavobacteriaceae; *Maribacter* | BD2-11 terrestrial group |
| Kiloniellaceae | - | NS11-12 marine group | Desulfobacteraceae; *Desulfococcus* |
| Woeseiaceae ;*Woeseia* | - | Thiotrichaceae; *Cocleimonas* | Desulfobulbaceae |
| Nitrosococcaceae ;*AqS1* | - | Thiovulaceae; *Sulfurimonas* | PHOS-HE36 |
| SAR324 clade  (Marine group B) | - | Unknown Family; *Marinicella* | SBR1031 |
| Subgroup 21 | - | Actinobacteria | Schekmanbacteria |
| Latescibacteria | - | Alteromonadaceae | WCHB1-41 |
| Magnetospiraceae | - | Anaerolineaceae | - |
| Nitrospiraceae ;Nitrospira | - | Burkholderiaceae; *Polaromonas* | - |
| OM190 | - | Clostridiales | - |
| PAUC43f marine benthic group | - | Colwelliaceae | - |
| Subgroup 6 | - | Crocinitomicaceae | - |
| Subgroup 9 | - | Desulfobulbaceae | - |

| **A** | **B** | **C** | **D** |
| --- | --- | --- | --- |
| Bacteriap25 | - | Desulfuromonadales | - |
| Cyclobacteriaceae | - | EPR3968-O8a-Bc78 | - |
| KI89A clade | - | Flavobacteriaceae; *Arcticiflavibacter* | - |
| *S085* | - | Flavobacteriaceae; *Gaetbulibacter* | - |
| SAR202 | - | Flavobacteriaceae; *Maritimimonas* | - |
| BD2-11 terrestrial group | - | Flavobacteriaceae; *Muriicola* | - |
| Chloroflexi | - | Methylophilaceae; *Methylotenera* | - |
| Phycisphaeraceae; Urania-1B-19 marine sediment group | - | Nitrincolaceae | - |
| Subgroup 26 | - | Nitrosococcaceae; *Cm1-21* | - |
| Dadabacteriales | - | Rhodobacteraceae; Roseobacter clade NAC11-7 lineage | - |
| *EPR3968-O8a-Bc78* | - | Rubritaleaceae; *Persicirhabdus* | - |
| *JG30-KF-CM66* | - | Sphingomonadaceae | - |
| Kiloniellaceae | - | Spirochaetaceae | - |
| MBNT15 | - | Spirosomaceae; *Taeseokella* | - |
| Pla3 lineage | - | - | - |
| Planctomycetes | - | - | - |
| Rhodobacteraceae | - | - | - |
| Rhodothermaceae | - | - | - |
| Saprospiraceae | - | - | - |
| Scalinduaceae; *Scalindua* | - | - | - |
| Sneathiellaceae; *AT-s3-44* | - | - | - |
| TK17 | - | - | - |
| VadinHA49 | - | - | - |
| *67-14* | - | - | - |
| AT-s2-59 | - | - | - |

| **A** | **B** | **C** | **D** |
| --- | --- | --- | --- |
| C86 | - | - | - |
| CCM11a | - | - | - |
| DEV007 | - | - | - |
| Eel-36e1D6 | - | - | - |
| Flavobacteriaceae | - | - | - |
| Gemmatimonadaceae | - | - | - |
| *KD4-96* | - | - | - |
| KF-JG30-B3 | - | - | - |
| Kiloniellaceae; *Limibacillus* | - | - | - |
| Latescibacteraceae | - | - | - |
| OM182 clade | - | - | - |
| P9X2b3D02 | - | - | - |
| Phycisphaeraceae | - | - | - |
| Rhizobiales Incertae Sedis; *Anderseniella* | - | - | - |
| Sandaracinaceae | - | - | - |
| Solibacteraceae (Subgroup 3) | - | - | - |
| Subgroup 7 | - | - | - |
| TRA3-20 | - | - | - |
| UBA10353 marine group | - | - | - |
